# Intercellular transfer of activated STING triggered by RAB22A-mediated non-canonical autophagy promotes antitumor immunity

**DOI:** 10.1038/s41422-022-00731-w

**Published:** 2022-10-24

**Authors:** Ying Gao, Xueping Zheng, Boyang Chang, Yujie Lin, Xiaodan Huang, Wen Wang, Shirong Ding, Weixiang Zhan, Shang Wang, Beibei Xiao, Lanqing Huo, Youhui Yu, Yilin Chen, Run Gong, Yuanzhong Wu, Ruhua Zhang, Li Zhong, Xin Wang, Qiuyan Chen, Song Gao, Zhengfan Jiang, Denghui Wei, Tiebang Kang

**Affiliations:** 1 Sun Yat-sen University Cancer Center, State Key Laboratory of Oncology in South China, Collaborative Innovation Center for Cancer Medicine, Guangzhou, Guangdong China; 2 Department of Interventional Radiology, The Third Affiliated Hospital of Sun Yat-sen University, Guangzhou, Guangdong China; 3 Department of Abdominal Oncology, The Cancer Center of the Fifth Affiliated Hospital of Sun Yat-sen University, Zhuhai, Guangdong China; 4 Key Laboratory of Cell Proliferation and Differentiation of the Ministry of Education, School of Life Sciences, Peking University, Beijing, China

**Keywords:** Macroautophagy, Endosomes, Multivesicular bodies, Small GTPases, Cancer microenvironment

## Abstract

STING, an endoplasmic reticulum (ER) transmembrane protein, mediates innate immune activation upon cGAMP stimulation and is degraded through autophagy. Here, we report that activated STING could be transferred between cells to promote antitumor immunity, a process triggered by RAB22A-mediated non-canonical autophagy. Mechanistically, RAB22A engages PI4K2A to generate PI4P that recruits the Atg12–Atg5–Atg16L1 complex, inducing the formation of ER-derived RAB22A-mediated non-canonical autophagosome, in which STING activated by agonists or chemoradiotherapy is packaged. This RAB22A-induced autophagosome fuses with RAB22A-positive early endosome, generating a new organelle that we name Rafeesome (RAB22A-mediated non-canonical autophagosome fused with early endosome). Meanwhile, RAB22A inactivates RAB7 to suppress the fusion of Rafeesome with lysosome, thereby enabling the secretion of the inner vesicle of the autophagosome bearing activated STING as a new type of extracellular vesicle that we define as R-EV (RAB22A-induced extracellular vesicle). Activated STING-containing R-EVs induce IFNβ release from recipient cells to the tumor microenvironment, promoting antitumor immunity. Consistently, RAB22A enhances the antitumor effect of the STING agonist diABZI in mice, and a high RAB22A level predicts good survival in nasopharyngeal cancer patients treated with chemoradiotherapy. Our findings reveal that Rafeesome regulates the intercellular transfer of activated STING to trigger and spread antitumor immunity, and that the inner vesicle of non-canonical autophagosome originated from ER is secreted as R-EV, providing a new perspective for understanding the intercellular communication of organelle membrane proteins.

## Introduction

The cGAS-STING signaling pathway plays an important role in innate immunity. STING, an endoplasmic reticulum (ER) transmembrane protein, is activated by its natural ligand cGAMP generated by cGAS, which recruits TANK-binding kinase 1 (TBK1) to activate interferon regulatory factor 3 (IRF3) through phosphorylation.^1^ This pathway induces the production of various cytokines including type I interferons (IFNs).^2^ The STING pathway recently emerges as a key regulator in cancer immunity and is being explored as a potential therapeutic target.^3^ STING agonists or cGAMP has a strong antitumor effect in vivo, but preclinical and clinical data have shown that the role of STING agonists in systemic drug administration is limited.^4^ In fact, cGAMP produced by tumor cells can be transferred to NK cells in the tumor microenvironment, which activates STING in these NK cells to execute antitumor immunity.^5^ However, cGAMP is highly unstable since it can be hydrolyzed by ectonucleotide pyrophosphatase/phosphodiesterase 1,^6^ resulting in a suboptimal therapeutic activity. Therefore, how the STING pathway can be better utilized as a therapeutic target still remains a challenge.

It has been reported that activated STING transmits downstream signals and is degraded through autophagy.^7,8^ It has been generally thought that ER-resident proteins without signal peptides are non-secreted proteins, and they are frequently used as exclusion markers of exosomes.^9^ Therefore, it is puzzling whether STING can be transported into extracellular vesicles (EVs).

EVs are present in biological fluids and function in intercellular communication, allowing cells to exchange proteins, lipids, genetic materials, amino acids, and metabolites.^10–12^ EVs are a heterogeneous group of cell-derived membranous structures mainly comprising exosomes and microvesicles (MVs), which originate from the endosomal system and are shed from the plasma membrane, respectively.^13,14^ Thus, it is well documented that exosomes and MVs bear many cell membrane proteins.^12,15^ Exosomes are generated as intraluminal vesicles (ILVs) within the lumen of endosomes during their maturation into multivesicular endosomes (MVEs) and secreted by the fusion of MVEs with the plasma membrane.^14,16^ The formation of ILVs by the inward budding of MVEs is mostly mediated by the Syntenin-Alix-ESCRT-III or RAB31-FLOTs machinery.^17,18^

Canonical autophagy, a highly conserved process of cellular component self-digestion essential for cell homeostasis and adaptation to stress,^19^ plays crucial roles in various physiological and pathological processes by either selectively or non-selectively degrading substrates, including protein aggregates, damaged mitochondria, ER, lipid droplets, and intracellular pathogens.^20–22^ The conserved machinery for canonical autophagosome formation contains two major initiation complexes: the ULK1 complex and the class III PI3-kinase complex I that generates PI3P; the generation of PI3P is an essential early event in canonical autophagy initiation.^23^ However, increasing evidence supports the presence of non-canonical autophagy that is characterized by the shared usage of the autophagy machinery and distinct components that function in multiple different scenarios.^24,25^ Over the past decade, the reported functions of non-canonical autophagy do not involve “self-eating” technically. One of the non-canonical autophagy processes was named secretory autophagy that facilitates unconventional secretion of the cytosolic cargo such as Acb1,^26,27^ IL-1β,^28^ and lysozyme.^29^

In this report, we identified a new organelle, Rafeesome (RAB22A-mediated non-canonical autophagosome fused with early endosome), and found that activated STING is packaged into such autophagosomes and secreted as cargo within a new type of EV, R-EV (RAB22A-induced extracellular vesicle). Our work reveals that Rafeesome regulates the intercellular transfer of activated STING, and that activated STING-containing EVs induce antitumor immunity in recipient cells.

## Results

### EVs containing activated STING induce IFNβ expression in recipient cells

**Fig. 1:**
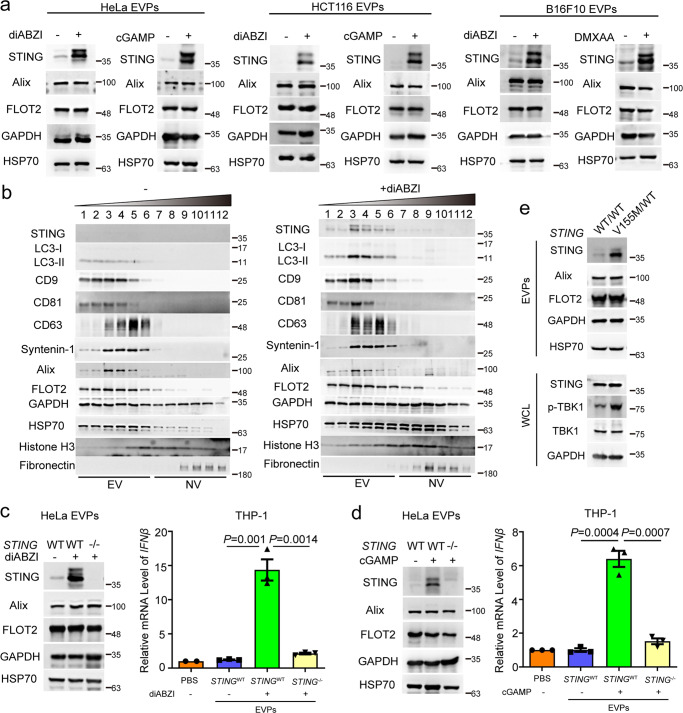
EVs containing activated STING induce IFNβ expression in recipient cells. **a** Western blot analysis of EVPs isolated from HeLa, HCT116, and B16F10 cells treated with or without 10 µM diABZI, 1 µM cGAMP, or 50 µg/mL DMXAA for 48 h. **b** Density gradient fractionation of EVPs derived from HeLa cells treated with or without diABZI. After flotation of the sample in high-resolution iodixanol gradients, equal volumes of each fraction were loaded on SDS-PAGE gels, and membranes were blotted with the indicated antibodies. NV, non-vesicular. **c**, **d** THP-1 cells were collected after 24-h exposure to EVPs that were derived from wild-type and *STING*^−/−^ HeLa cells treated with or without diABZI (**c**) and cGAMP (**d**), respectively. The mRNA level of *IFNβ* was then quantified. *P* values were calculated by Student’s *t*-test. **e** Western blot analysis of the WCL and the corresponding isolated EVPs derived from wild-type HeLa cells or HeLa cells expressing STING^V155M/WT^.

To investigate whether STING is present on EVs, we collected EVs and particles (EVPs, 100 K pellet)^30,31^ from various tumor cells treated with or without STING agonists.^32,33^ Interestingly, STING agonist treatment effectively activated the STING pathway in these cell lines (Supplementary information, Fig. S1a–c) and promoted activated STING to be packaged into EVPs. In contrast, STING was hardly detected in EVPs derived from untreated cells (Fig. 1a and Supplementary information, Fig. S1d–i). High-resolution density gradient fractionation was employed to separate the EVs and non-vesicular particles in EVPs. STING was highly enriched in the EV fraction pools obtained from diABZI-treated cells, as indicated by the well-known EV markers, such as CD9, CD81, CD63, Syntenin-1, Alix, FLOT2, GAPDH, and HSP70 (Fig. 1b). These results indicate that activated STING is detectable in EVs derived from a variety of cells upon stimulation.

Importantly, the production of IFNβ was detected in human monocyte THP-1 cells incubated with EVPs from diABZI-treated HeLa cells (Supplementary information, Fig. S1j), but not from diABZI-treated STING-deleted HeLa cells (*STING*^−/−^) (Fig. 1c, d). When we constructed stable cell lines with constitutively active STING mutations (STING^V155M^ and STING^R281Q^), a large number of cells died at the beginning, but a small number of cells with low expression of active STING could normally grow in cell culture; then these survived cells were used to produce EVPs. Consistently, EVPs derived from HeLa cells stably expressing these two active STING mutants, but not an empty vector, induced IFNβ expression in THP-1 cells (Supplementary information, Fig. S1k). The induction of IFNβ by EVPs was not changed in THP-1 cells with knockdown of cGAS (Supplementary information, Fig. S1l). These results indicate that the induction of IFNβ by EVPs in THP-1 cells is caused by activated STING, but not by DNA, in EVPs. Notably, we generated heterozygous STING^V155M/WT^ knock-in HeLa cells, and found that significantly more STING entered EVPs in STING^V155M/WT^ cells than in WT cells (Fig. 1e and Supplementary information, Fig. S1m). Similar results were obtained using other donor cancer cells such as HCT116 and MDA-MB-231 cells (Supplementary information, Fig. S1n), and other recipient cells, such as mouse bone marrow-derived macrophages (BMDMs) and mouse fibroblast L929 cells (Supplementary information, Fig. S1o). Collectively, these results demonstrate that activated STING is sorted into EVs from various cells and can directly induce IFNβ expression in recipient cells.

### Activated STING-containing EVs execute antitumor activity

**Fig. 2:**
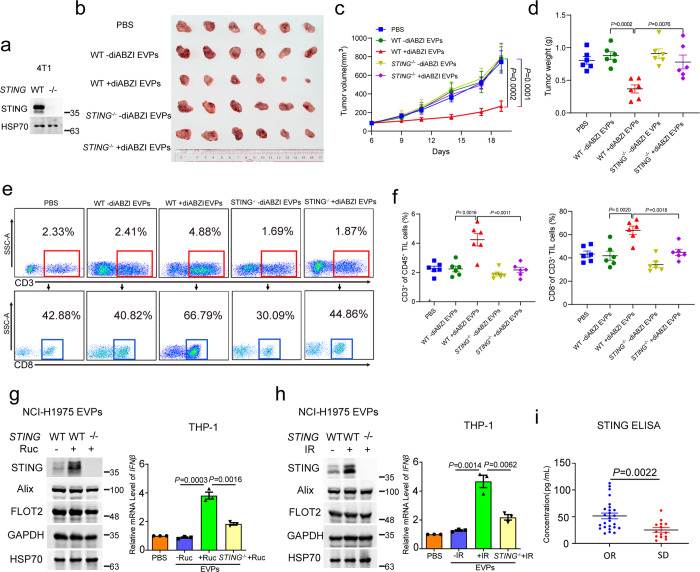
EVs containing activated STING execute antitumor activity. **a** Knockout efficiency of *STING* in 4T1 cells shown by western blot analysis. **b**–**d** BALB/c mice were implanted subcutaneously with mouse 4T1 tumor cells for a week followed by tail vein injection of PBS or 5 μg EVPs derived from 4T1 cells three times a week. The xenografts were excised (**b**); tumor volume was monitored three times a week, *P* values were calculated by two-way ANOVA (**c**). Tumor weight on day 19 after transplantation was shown, and *P* value was calculated by Student’s *t*-test (**d**). *n* = 6. The data were presented as means ± SEM. **e**, **f** FACS analysis showing the infiltration of both CD3^+^ and CD8^+^ T cells in BALB/c mice bearing subcutaneous 4T1 tumors. *P* values were calculated by Student’s *t*-test. **g**, **h** THP-1 cells were collected after 24-h exposure to EVPs harvested from conditioned media of wild-type and *STING*^−/−^ NCI-H1975 cells stimulated with 25 μM Ruc (**g**) or 8 Gy IR (**h**). The mRNA level of *IFNβ* was then quantified. *P* values were calculated by Student’s *t*-test. **i** Serum STING concentrations in NPC patients with different responses to chemoradiotherapy. *P* value was calculated by Student’s *t*-test. *n* = 26 for OR, and *n* = 14 for SD. OR, objective response; SD, stable disease.

Since IFNβ secreted by macrophages promotes the infiltration of T cells that kill tumor cells in tumor tissues,^34^ the role of EVPs containing activated STING in tumor growth in mice was investigated. 4T1 cells were subcutaneously injected into the right inguinal area of BALB/c mice for 6 days, and these mice were then administered with PBS or EVPs via tail vein injection. As shown in Fig. 2, EVPs from diABZI-treated 4T1 cells significantly reduced both tumor size and tumor weight (Fig. 2b–d) and increased the infiltration of both CD3^+^ and CD8^+^ T cells in BALB/c mice bearing subcutaneous 4T1 tumors (Fig. 2e, f). These effects were mostly diminished using EVPs derived from diABZI-treated *STING*^−/−^ 4T1 cells (Fig. 2a–f). These results indicate that activated STING-containing EVs execute antitumor immunity to suppress tumor growth in mice.

It has been reported that STING can be activated in cancer cells treated by chemoradiotherapy,^35,36^ and we also found that the STING-TBK1 pathway was activated in NCI-H1975 cells treated with various chemotherapeutic drugs, including cisplatin (DDP), adriamycin (ADR), rucaparib (Ruc), hydroxyurea (HU) and 5-fluorouracil (5-FU), as well as irradiation (IR) (Supplementary information, Fig. S2a, b). Furthermore, the EVPs derived from NCI-H1975 cells treated with either Ruc or IR could also stimulate IFNβ expression in THP-1 recipient cells, which was diminished in STING-deleted NCI-H1975 (*STING*^−/−^) cells (Fig. 2g, h). More importantly, STING was also detected in serum EVPs from nasopharyngeal cancer (NPC) patients after chemotherapy and/or radiotherapy by western blot (Supplementary information, Fig. S2c). As shown in Fig. 2i, during the course of chemotherapy and/or radiotherapy, STING levels in serum of NPC patients with objective response were higher than those in the serum of patients with stable disease as shown by ELISA, indicating that the STING level in serum positively correlates with the sensitivity of NPC patients to chemoradiotherapy.

### RAB22A controls the formation of activated STING-containing MVB-like structures and the secretion of activated STING-containing EVs

Next, using a library containing 62 constitutively active RAB GTPases that we have recently generated,^18^ the distribution patterns of STING^V155M^-HA with Flag-tagged RAB GTPases were investigated (Supplementary information, Fig. S3a), because RAB proteins regulate vesicle formation, transport, and fusion.^37–39^ We found that STING^V155M^-HA was localized on ILVs in multivesicular body (MVB)-like structures induced by RAB5 (RAB5A^Q79L^, RAB5B^Q79L^, RAB5C^Q113L^) and RAB22A^Q64L^ (Supplementary information, Fig. S3b). However, MVB-like structures driven by RAB5A^Q79L^ co-localized substantially with Lyso-Tracker (Supplementary information, Fig. S3c), indicating that these MVB-like structures driven by RAB5 tend to be acidic, which are destined for degradation.

**Fig. 3:**
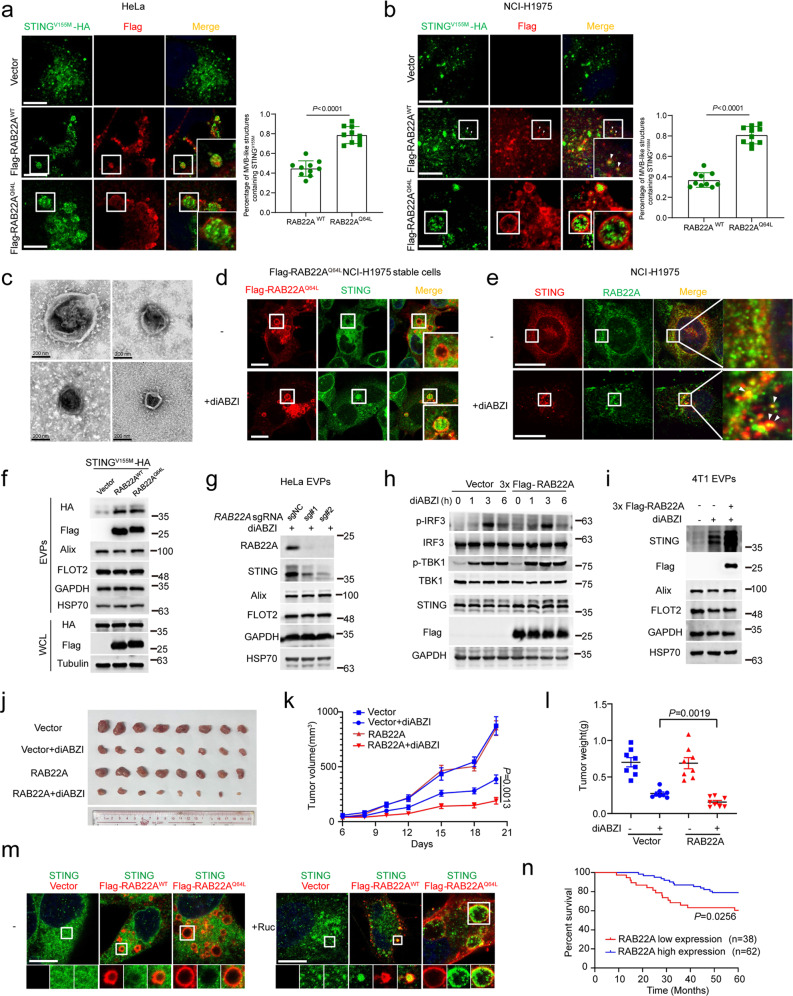
RAB22A enhances the antitumor effect of diABZI and predicts prognosis in NPC patients treated with chemoradiotherapy. **a**, **b** Immunofluorescence analysis of STING^V155M^-HA (green) and Flag (red) in the indicated stable HeLa (**a**) or NCI-H1975 (**b**) cells transiently expressing STING^V155M^-HA. Percentage of MVB-like structures containing STING^V155M^ was quantified on the right. *P* values were calculated by Student’s *t*-test. White arrowheads denote co-localization. *n* = 10 fields (each field has at least 8 cells that meet statistical requirements). Scale bar, 10 μm. **c** Electron microscopy images showed the structure of L-Flag-STING^V155M^-GFP EVs. Scale bar, 200 nm. **d** Immunofluorescence analysis of STING (green) and Flag-RAB22A^Q64L^ (red) in stable Flag-RAB22A^Q64L^ NCI-H1975 cells treated with or without 1 µM diABZI for 1 h. Scale bar, 10 μm. **e** Immunofluorescence analysis of STING (red) and RAB22A (green) in NCI-H1975 cells treated with or without 1 µM diABZI for 1 h. Arrowheads indicate the co-localization of STING with RAB22A. Scale bar, 10 μm. **f** Western blot analysis of EVPs and WCL derived from the indicated stable 3× Flag-RAB22A^WT^ or 3× Flag-RAB22A^Q64L^ HeLa cells simultaneously and stably expressing STING^V155M^-HA. **g** Western blot analysis of EVPs derived from *RAB22A*-KO HeLa cells treated with 10 µM diABZI for 48 h. **h** Western blot analysis of the WCL of the indicated stable 4T1 cells at the indicated time points after treatment with 10 µM diABZI. **i** Western blot analysis of the EVPs from the indicated stable 4T1 cells treated with or without 10 µM diABZI. **j**–**l** Dissected tumors (**j**), tumor growth curve (**k**), and tumor weight (**l**) for xenograft experiments. The stable 4T1 cells were inoculated orthotopically into BALB/c mice for 6 days as indicated, and the mice were then treated with or without 1.5 mg/kg diABZI three times per week by intravenous tail injection as indicated for another 14 days. Visible tumors were measured every 2 or 3 days. *P* value of tumor growth curve was calculated by two-way ANOVA, and *P* value of tumor weight was calculated by Student’s *t*-test. *n* = 8. The data are presented as the means ± SEM. **m** Immunofluorescence analysis of STING (green), Flag (red), and DAPI (blue) in the indicated NCI-H1975 stable cells treated with or without 25 µM Ruc for 48 h. Scale bar, 10 μm. **n** Kaplan–Meier survival analyses of NPC cases separated into two groups by the median expression levels of RAB22A as indicated by RAB22A staining. *P* value was calculated by log-rank test.

Notably, RAB22A^WT^ also induced the formation of MVB-like structures containing STING^V155M^-HA, and these MVB-like structures were smaller than those induced by RAB22A^Q64L^ in HeLa cells and NCI-H1975 cells (Fig. 3a, b). To verify the existence of activated STING-containing EVs, we constructed an L-Flag-STING^V155M^-GFP plasmid, in which a Flag tag was inserted into the luminal domain and GFP was tagged at the C-terminus of STING, according to the structure of the STING protein.^40^ Cells with strong L-Flag-STING^V155M^-GFP signals were sorted by flow cytometry, and the EVPs derived from these cells were collected and exposed to anti-Flag magnetic beads. Then, an L-Flag-STING^V155M^-GFP EV suspension was obtained through elution with Flag peptides. The spherical structures of L-Flag-STING^V155M^-GFP EVs were observed by transmission electron microscopy (TEM) as shown in Fig. 3c. NanoSight nanoparticle tracking analysis revealed that the peak diameter of L-Flag-STING^V155M^-GFP EVs was ~200 nm, which was much larger than that of EVs (~100 nm in diameter) derived from vector-expressing cells (Supplementary information, Fig. S3d), indicating that activated STING-containing EVs may not be classical exosomes.

More importantly, endogenous STING was localized on ILVs in the MVB-like structures driven by RAB22A^Q64L^ in NCI-H1975 cells treated with diABZI (Fig. 3d), and the co-localization of STING with RAB22A at their endogenous levels was induced by diABZI treatment in NCI-H1975 cells (Fig. 3e). STING^V155M^ or STING^R281Q^ levels in EVPs, but not their intracellular levels, could be increased by both RAB22A^WT^ and RAB22A^Q64L^ (Fig. 3f and Supplementary information, Fig. S3e). Accordingly, depletion of RAB22A significantly reduced endogenous STING levels in the EVPs derived from both HeLa and NCI-H1975 cells treated with diABZI (Fig. 3g and Supplementary information, Fig. S3f). In addition, RAB22A increased endogenous STING levels in EVPs derived from 4T1 cells treated with diABZI, while the activity of intracellular STING signaling remained unaffected under this condition (Fig. 3h, i). Moreover, RAB22A could enhance the antitumor effect of diABZI on 4T1 tumors in an orthotopic mouse model (Fig. 3j–l). Taken together, these results show that RAB22A controls the formation of activated STING-containing MVB-like structures and the secretion of activated STING-containing EVs.

Consistently, the localization of endogenous STING in MVB-like structures was clearly observed in NCI-H1975 cells stably expressing RAB22A^WT^ or RAB22A^Q64L^ in response to Ruc treatment (Fig. 3m), suggesting that cancer patients bearing high RAB22A levels in tumors may benefit from chemoradiotherapy, as the production of IFNβ was induced in THP-1 cells incubated with EVPs derived from NCI-H1975 cells treated with Ruc or IR (Fig. 2g, h). It was the case that the overall survival rate was significantly higher in patients with NPC bearing high levels of RAB22A after chemoradiotherapy compared with those having low levels of RAB22A (Fig. 3n and Supplementary information, Fig. S3g), indicating that higher RAB22A levels predict better prognoses for patients with NPC after chemoradiotherapy.

### Activated STING is packaged into MVB-like structures dependent on non-canonical autophagosomes whose formation is driven by RAB22A

We next investigated the mechanism by which activated STING-containing ILVs are formed in MVB-like structures whose formation is driven by RAB22A. As shown in Supplementary information, Fig. S4a, knockout of *SDCBP* did not affect the localization of activated STING in MVB-like structures induced by RAB22A^Q64L^ but blocked the entry of CD63 into these structures, as expected.^41^ The lipid raft-associated proteins FLOT1 and FLOT2 were localized on the outer membrane of the MVB-like structures, but they did not co-localize with activated STING on ILVs in these MVB-like structures (Supplementary information, Fig. S4b). These results indicate that the MVB-like structures induced by RAB22A^Q64L^ may represent a novel type of MVB whose formation depends on neither the Syntenin-Alix-ESCRT-III nor RAB31-FLOTs exosome pathway.^18,42^

**Fig. 4:**
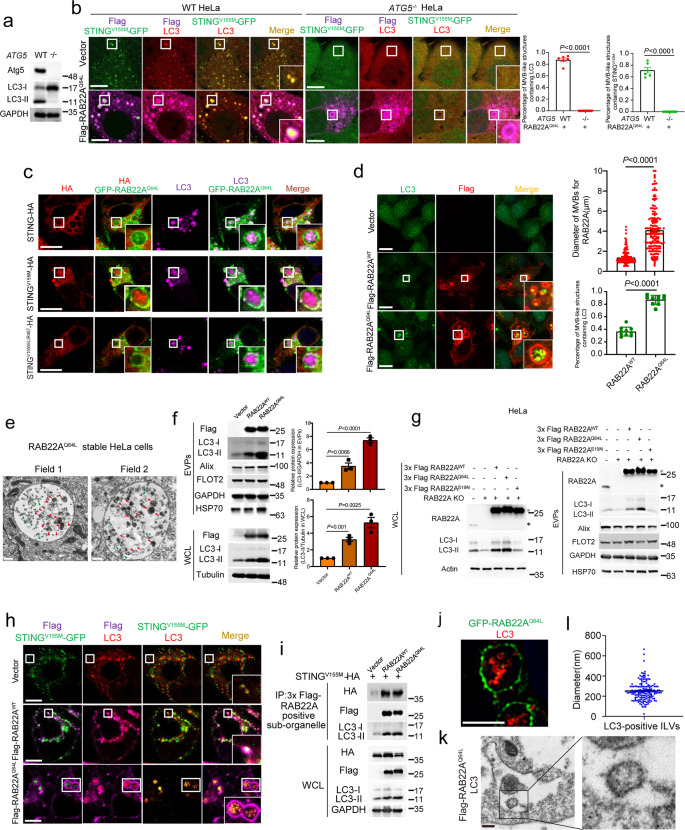
Activated STING is packaged into MVB-like structures whose formation is driven by RAB22A. **a** Knockout efficiency of *ATG5* shown by western blot analysis. **b** Immunofluorescence analysis of LC3 (red), Flag-RAB22A^Q64L^ (magenta), and transiently transfected STING^V155M^-GFP (green) in wild-type or *ATG5*-KO HeLa cells. Percentages of MVB-like structures containing LC3 and STING^V155M^ were quantified respectively on the right. *P* values were calculated by Student’s *t*-test. *n* = 6 fields. Scale bar, 10 μm. **c** Immunofluorescence analysis of LC3 (magenta), HA (red), and GFP-RAB22A^Q64L^ (green) in the indicated HeLa cells stably expressing STING-HA, STING^V155M^-HA or STING^V155M/LIR467^-HA. Scale bar, 10 μm. **d** Immunofluorescence analysis of LC3 (green) and Flag (red) in the indicated stable HeLa cells. Percentages of MVB-like structures containing LC3 (*n* = 10 fields) and diameters of RAB22A-positive MVBs (*n* = 150) were quantified on the right. *P* values were calculated by Student’s *t*-test. Scale bar, 10 μm. **e** Intracellular structures were observed by electron microscopy in the indicated stable HeLa cells. Scale bar, 500 nm. **f** Western blot analysis of WCL and EVPs derived from the 3× Flag-RAB22A^WT^ and 3× Flag-RAB22A^Q64L^ stable HeLa cells. Relative protein expression was quantified on the right. *P* values were calculated by Student’s *t*-test. **g** Western blot analysis of WCL and EVPs derived from the *RAB22A*-KO HeLa cells reintroduced with 3× Flag-RAB22A^WT^, 3× Flag-RAB22A^Q64L^, or 3× Flag-RAB22A^S19N^ as indicated. * represents the bands of RAB22A; ※ represents the bands of 3× Flag-RAB22A. **h** Immunofluorescence analysis of LC3 (red), Flag (magenta), and STING^V155M^-GFP (green) in the indicated stable HeLa cells transiently expressing STING^V155M^-GFP. Scale bar, 10 μm. **i** Western blot analysis of WCL and immunoprecipitated RAB22A-positive sub-organelles derived from the indicated stable HeLa cells. **j** Immunofluorescence analysis of GFP-RAB22A^Q64L^ (green) and LC3 (red) in GFP-RAB22A^Q64L^ stable HeLa cells using SIM. Scale bar, 5 μm. **k** Immunoelectron microscopy showing LC3 localization in the indicated stable HeLa cells. Scale bar, 200 nm. **l** The diameters of LC3-positive ILVs were calculated. *n* = 156.

Activated STING can induce autophagy,^8^ and STING has the LC3-interaction region (LIR) motif through which it binds LC3.^7,43^ Therefore, we explored whether autophagy regulates the packaging of activated STING into MVB-like structures. Co-localization of activated STING with LC3 in the MVB-like structures whose formation was driven by RAB22A^Q64L^ was clearly observed, and neither activated STING nor LC3 was localized in these MVB-like structures in cells with the autophagy-essential gene *ATG5, ATG7* or *ATG16L1* knockout (Fig. 4a, b and Supplementary information, Fig. S4c, d), indicating that autophagosome formation is required for the packaging of activated STING into MVB-like structures. In addition, STING^V155M^-HA and STING^R281Q^-HA expression resulted in a higher level of LC3-II compared with wild-type STING-HA in HeLa stable cells (Supplementary information, Fig. S4e). Strikingly, both STING^V155M^ and STING^R281Q^, but not wild-type STING, bound LC3-II (Supplementary information, Fig. S4e), and this interaction was dependent on the LIR motif, as the LIR-mutant STING^V155M/LIR467^ failed to bind V5-LC3 (Supplementary information, Fig. S4f). STING^V155M^-HA, but not its wild-type (STING-HA) and LIR-mutant STING^V155M/LIR467^, along with LC3 could be packaged into MVB-like structures driven by RAB22A^Q64L^ (Fig. 4c). Upon diABZI or cGAMP stimulation, STING-HA became active and bound to LC3-II, and more LC3-II was detected (Supplementary information, Fig. S4g, h), consistent with our previous observation that activated STING, rather than the inactive form, was secreted into EVs (Fig. 1a and Supplementary information, Fig. S1e–i).

However, as shown in Supplementary information, Fig. S4i, j, the packaging of LC3 into the MVB-like structures whose formation was driven by RAB22A^Q64L^ was not affected in cells with depletion of *SAR1A*, a gene required for activated STING-induced autophagy^8^ (Supplementary information, Fig. S4k). Consistently, the packaging of activated STING into the MVB-like structures was even slightly increased in *SAR1A*-depeleted cells, which might be due to the inhibition of STING degradation through STING-induced autophagy. Together, these results suggest that a new type of non-canonical autophagy mediated by RAB22A may be harnessed to package activated STING into MVB-like structures. Indeed, RAB22A^WT^ and RAB22A^Q64L^ induced the formation of MVB-like structures containing LC3 in HeLa cells (Fig. 4d) and NCI-H1975 cells (Supplementary information, Fig. S4l). In addition, many MVB-like structures could be observed using electron microscopy in cells overexpressing RAB22A^Q64L^. These MVB-like structures seemed to contain two types of ILVs: one may be the classical ILVs with smaller sizes (Fig. 4e, green arrowheads), and the other with larger sizes may be the inner vesicles of RAB22A-induced non-canonical autophagosomes (Fig. 4e, red arrowheads).

Accordingly, overexpression of RAB22A^WT^ or RAB22A^Q64L^ increased LC3-II levels in both whole-cell lysates (WCL) and EVPs. This LC3-II-increasing effect induced by RAB22A^Q64L^ was much stronger than that induced by RAB22A^WT^ (Fig. 4f), and was not affected by *SAR1A* knockout (Supplementary information, Fig. S4m). The packaging of LC3 into the MVB-like structures induced by RAB22A^Q64L^ was not affected by *STING* deletion either (Supplementary information, Fig. S4n). Moreover, autophagic flux inhibitors Bafilomycin A1 (BafA1) and chloroquine (CQ), which block autophagosome–lysosome fusion, further promoted LC3-II accumulation induced by overexpression of RAB22A^WT^ or RAB22A^Q64L^, indicating that RAB22A promoted LC3-II formation (Supplementary information, Fig. S4o). The LC3-II levels in both WCL and EVPs were decreased when endogenous *RAB22A* was depleted (Fig. 4g and Supplementary information, Fig. S4p). The LC3-II levels were restored in the *RAB22A*-knockout HeLa cells reintroduced with RAB22A^WT^ and RAB22A^Q64L^, but not the inactive form RAB22A^S19N^ that was locked to GDP (Fig. 4g). These results suggest that the formation of the LC3 puncta driven by RAB22A is independent of the formation of autophagosomes driven by STING. Indeed, STING^V155M^ and LC3 were co-localized in MVB-like structures whose formation was driven by RAB22A^WT^ and RAB22A^Q64L^, as shown by immunofluorescence assay (Fig. 4h). Consistently, STING^V155M^ and LC3-II were enriched in the MVB-like structures induced by RAB22A, which were isolated by the sub-organelle extraction method (Fig. 4i). Using super-resolution structured illumination microscopy (SIM), ILVs bearing LC3 were clearly observed in RAB22A^Q64L^-induced MVB-like structures (Fig. 4j). In addition, as shown in immunoelectron microscopy (IEM) images, LC3 was localized on the ILVs in MVB-like structures induced by RAB22A^Q64L^ (Fig. 4k), and the diameters of these ILVs mainly ranged from 100 to 400 nm (Fig. 4l).

Taken together, these results indicate that activated STING is packaged into a type of non-canonical autophagosome, which is subsequently converted into an MVB-like structure. Therefore, we designated the ILVs derived from the inner vesicles of non-canonical autophagosomes in these MVB-like structures whose formation is driven by RAB22A as R-EVs (RAB22A-induced EVs) when they are eventually secreted, and we propose that R-EVs may represent a novel type of EV.

### The RAB22A-mediated non-canonical autophagosome fuses with early endosome developing into Rafeesome

**Fig. 5:**
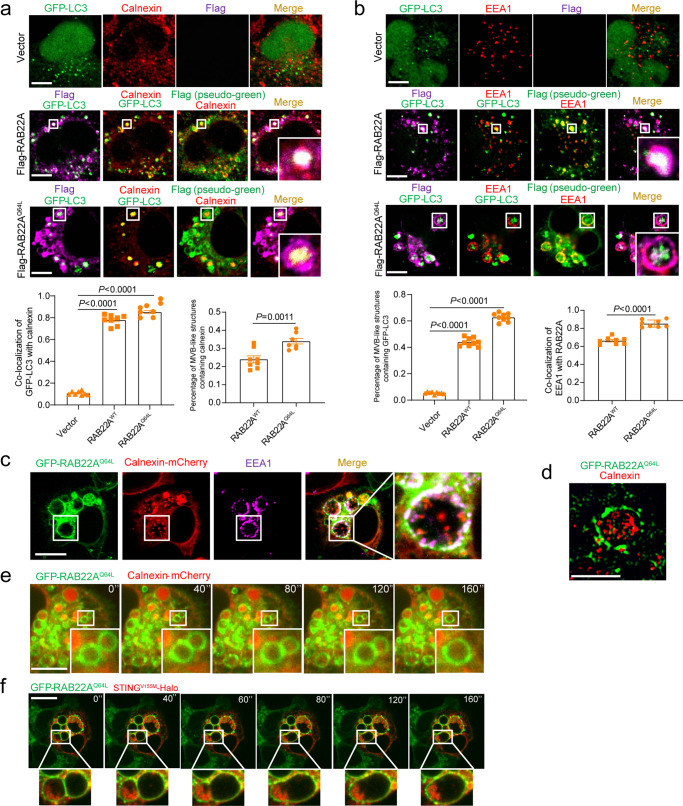
RAB22A-mediated non-canonical autophagosomes originate from ER and fuse with RAB22A-positive early endosomes to generate Rafeesomes. **a** Immunofluorescence analysis of the ER marker calnexin (red), Flag (magenta or pseudo-green as indicated) and GFP-LC3 (green) in the indicated stable HeLa cells transiently expressing GFP-LC3. Co-localization of GFP-LC3 with calnexin and the percentage of MVB-like structures containing calnexin were quantified. *P* values were calculated by Student’s *t*-test. *n* = 8 fields. Scale bar, 10 μm. **b** Immunofluorescence analysis of the early endosome marker EEA1 (red), Flag (magenta or pseudo-green as indicated), and GFP-LC3 (green) in the indicated stable HeLa cells transiently expressing GFP-LC3. Percentage of MVB-like structures containing GFP-LC3 and co-localization of EEA1 with RAB22A were quantified. *n* = 8 fields. Scale bar, 10 μm. **c** Immunofluorescence analysis of GFP-RAB22A^Q64L^ (green), EEA1 (magenta), and calnexin-mCherry (red) in GFP-RAB22A^Q64L^ stable HeLa cells transiently expressing calnexin-mCherry. Scale bar, 10 μm. **d** Immunofluorescence analysis of GFP-RAB22A^Q64L^ (green) and calnexin (red) in GFP-RAB22A^Q64L^ stable HeLa cells using SIM. Scale bar, 5 μm. **e** Time-lapse images showing GFP-RAB22A^Q64L^ (green) and calnexin-mCherry (red) in living tet-on GFP-RAB22A^Q64L^ stable HeLa cells transiently expressing calnexin-mCherry treated with 50 ng/mL doxycycline (dox) (Supplementary information, Video S1). Scale bar, 10 μm. **f** Time-lapse images showing GFP-RAB22A^Q64L^ (green) and STING^V155M^-Halo (red) in living tet-on GFP-RAB22A^Q64L^ stable HeLa cells transiently expressing STING^V155M^-Halo and treated with 50 ng/mL dox (Supplementary information, Video S2). Scale bar, 10 μm.

Since the formation of LC3 puncta driven by RAB22A is independent of the formation of autophagosomes driven by activated STING (Fig. 4c and Supplementary information, Fig. S4i, j, m, n), we sought to examine the origin of the RAB22A-mediated non-canonical autophagosome. The MVB-like structures induced by RAB22A^Q64L^ were not related to mitochondria, the Golgi apparatus, or the ER–Golgi intermediate compartment (ERGIC), because LC3 was not co-localized with the corresponding markers Tim23, GM130, or ERGIC-53 in cells stably expressing RAB22A^Q64L^ (Supplementary information, Fig. S5a). Intriguingly, LC3 was co-localized with the ER marker calnexin, and both LC3 and calnexin were distributed on the ILVs in RAB22A-induced MVB-like structures (Fig. 5a). Supportively, another ER marker TRAM1 was also distributed on the ILVs in MVB-like structures driven by RAB22A^Q64L^ (Supplementary information, Fig. S5b). Brefeldin A (BFA), an inhibitor reported to suppress the formation of autophagosomes induced by activated STING by blocking the translocation of activated STING from ER to ERGIC,^8^ was used to treat the cells. However, treatment with BFA did not prevent STING^V155M^ from being packaged into the RAB22A^Q64L^-induced MVB-like structures (Supplementary information, Fig. S5c), suggesting that STING^V155M^ is synthesized in ER and may be partially drawn into the RAB22A-mediated autophagosome before its translocation to ERGIC and Golgi. Together, these results indicate that RAB22A-mediated non-canonical autophagosome originates from ER.

Notably, RAB22A^WT^ or RAB22A^Q64L^ co-localized with EEA1 on the surface of MVB-like structures that contained LC3 (Fig. 5b), and both RAB22A^Q64L^ and EEA1 were localized on the surface of MVB-like structures containing calnexin in cells stably expressing RAB22A^Q64L^ (Fig. 5c). Using SIM, ILVs bearing calnexin were clearly observed in MVB-like structures whose formation was driven by RAB22A^Q64L^ (Fig. 5d). Furthermore, dynamic real-time fluorescence imaging showed that non-canonical autophagosomes and/or MVB-like structures containing calnexin or STING^V155M^ fused with RAB22A-positive early endosomes in cells stably expressing RAB22A^Q64L^ (Fig. 5e, f and Supplementary information, Videos S1 and S2).

Taken together, these results demonstrate that the RAB22A-mediated non-canonical autophagosomes originate from ER, and fuse with RAB22A-positive early endosomes to become MVB-like structures. Therefore, for ease of description, we define the autophagosome–endosome fusion structure whose formation is mediated by RAB22A as Rafeesome, a new organelle that may be critical for the transfer of activated STING.

### RAB22A inactivates RAB7, enabling the secretion of inner vesicles of non-canonical autophagosomes as R-EVs from Rafeesomes

Using a tandem GFP-RFP-LC3 assay, we found that the red LC3 puncta induced by rapamycin (Rapa) were dominant, indicating that they are autolysosomes in which GFP is quenched by an acidic lysosome environment. However, the LC3 puncta were mainly yellow in cells overexpressing RAB22A, indicating that the RAB22A-mediated non-canonical autophagosomes and/or Rafeesomes did not fuse with lysosomes (Supplementary information, Fig. S6a). In fact, the fusion of autophagosomes with lysosomes depends on active RAB7 binding to its effector RILP.^44^ We showed that active RAB7 was reduced in cells overexpressing RAB22A^WT^ or RAB22A^Q64L^ (Supplementary information, Fig. S6b), and active RAB7 was increased in cells with *RAB22A* knockout (Supplementary information, Fig. S6c). Collectively, these results reveal that RAB22A may promote the secretion of R-EVs by suppressing the fusion of Rafeesomes with lysosomes through inactivating RAB7.

### RAB22A-mediated non-canonical autophagy depends on PI4P generated by PI4K2A

Finally, we explored how RAB22A was involved in non-canonical autophagosome formation. By knocking out genes essential for canonical autophagy, we found that the RAB22A^Q64L^-mediated non-canonical autophagy was dependent on Atg5, Atg7, and Atg16L1 but not FIP200, Beclin1, or WIPI2 (Supplementary information, Fig. S7a–f). Indeed, Refeesomes did contain LC3 in cells with *WIPI2* knockout but not in those with *ATG7* knockout (Supplementary information, Fig. S7g), and LC3 whose expression was induced by both RAB22A^WT^ and RAB22A^Q64L^ did not co-localize with DFCP1 or WIPI2 (Supplementary information, Fig. S7h, i). While Atg16L1, LC3, and RAB22A were co-localized, Atg16L1 did not enter Refeesomes induced by RAB22A^Q64L^, as shown in Supplementary information, Fig. S7j, possibly because Atg16L1 leaves autophagosome when it maturates.^45^

**Fig. 6:**
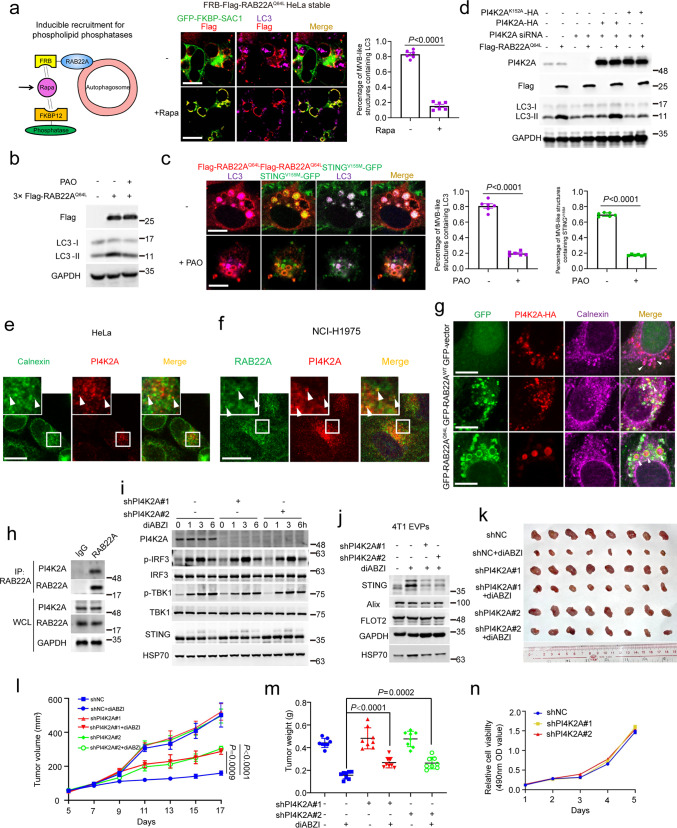
RAB22A-mediated non-canonical autophagy depends on PI4P generated by PI4K2A. **a** Left, schematic diagram showing inducible recruitment system for phosphatases: Rapa (1 μM, 6 h) induces the heterodimerization of FRB and FKBP12, thereby recruiting the phosphatase to RAB22A to hydrolyze its target phospholipid. Right, inducible translocation of the 4’ phosphatase (GFP-FKBP-SAC1) to FRB-Flag-RAB22A^Q64L^-induced Refeesomes. Immunofluorescence analysis of Flag (red), LC3 (magenta), and GFP-FKBP-SAC1 (green) in FRB-Flag-RAB22A^Q64L^ stable HeLa cells treated with or without 1 μM Rapa for 6 h. Percentage of MVB-like structures containing LC3 was quantified on the right. *P* value was calculated by Student’s *t*-test. *n* = 6 fields. Scale bar, 10 μm. **b** Western blot analysis of WCL from stable Flag-RAB22A^Q64L^ HeLa cells treated with the PI4K inhibitor PAO (5 μM) for 4 h. **c** Immunofluorescence analysis of Flag (red) and LC3 (magenta) with STING^V155M^-GFP (green) in stable Flag-RAB22A^Q64L^ HeLa cells transiently expressing STING^V155M^-GFP and treated with 5 μM PAO for 4 h. Percentages of MVB-like structures containing LC3 and STING^V155M^ were quantified respectively on the right. *P* values were calculated by Student’s *t*-test. *n* = 6 fields. Scale bar, 10 μm. **d** Western blot analysis of WCL from stable Flag-RAB22A^Q64L^ HeLa cells transfected with two different siRNAs targeting PI4K2A and reintroduced with WT PI4K2A or kinase-dead PI4K2A^K152A^. **e** Immunofluorescence analysis of endogenous calnexin (green) and PI4K2A (red) in HeLa cells. White arrowheads denote co-localization. Scale bar, 10 μm. **f** Immunofluorescence analysis of endogenous RAB22A (green) and PI4K2A (red) in NCI-H1975 cells. White arrowheads denote co-localization. Scale bar, 10 μm. **g** Immunofluorescence analysis of GFP (green), calnexin (magenta), and PI4K2A-HA (red) in the indicated stable HeLa cells transiently expressing PI4K2A-HA. White arrowheads denote co-localization. Scale bar, 10 μm. **h** Western blot analysis of WCL and immunoprecipitates from HeLa cells using an anti-RAB22A antibody. **i** Western blot analysis of the WCL of PI4K2A-knockdown stable 4T1 cells treated with 10 µM diABZI at the indicated time points. **j** Western blot analysis of the EVPs from PI4K2A-knockdown stable 4T1 cells treated with or without 10 µM diABZI. **k**–**m** Dissected tumors (**k**), tumor growth curve (**l**), and tumor weight (**m**) for xenograft experiments. The indicated stable 4T1 cells were inoculated orthotopically into BALB/c mice for 5 days, and the mice were then treated with or without 3 mg/kg diABZI three times per week by intravenous tail injection as indicated for another 12 days. Visible tumors were measured every 2 days. *P* value of tumor growth curve was calculated by two-way ANOVA, and *P* value of tumor weight was calculated by Student’s *t*-test. *n* = 8. The data are presented as the means ± SEM. **n** Cell viability was measured by the MTT assay in the indicated stable 4T1 cells.

PI3P is a conventional phosphoinositide binding to WIPI2, which subsequently recruits the Atg12–Atg5–Atg16L1 complex to generate LC3-II on phagophore.^46–48^ However, we found that the PI3K inhibitor 3-MA did not inhibit the increase in LC3-II expression induced by RAB22A^Q64L^, but diminished that induced by RAB1B^Q67L^ or RAB5A^Q79L^ (Supplementary information, Fig. S7k), indicating that PI3P did not participate in the RAB22A^Q64L^-mediated non-canonical autophagy initiation. PI4P and PI5P have also been reported to participate in autophagosome biogenesis.^49,50^ The membrane phospholipid composition of RAB22A^Q64L^-regulated non-canonical autophagosomes was investigated using an inducible recruitment system for phosphatases^51^ (Fig. 6a). The RAB22A^Q64L^-induced Rafeesomes still contained LC3 when inositol polyphosphate-5-phosphatase E (INPP5E) was directed to RAB22A^Q64L^ (Supplementary information, Fig. S7l). However, the enlarged RAB22A-positive endosomes did not contain LC3 when phosphatidylinositol-4-phosphate phosphatase (SAC1) was directed to RAB22A^Q64L^ (Fig. 6a), indicating that PI4P is required as the phosphoinositide component in the membrane for RAB22A-mediated non-canonical autophagosome formation. Indeed, the PI4K inhibitor PAO suppressed the increase in LC3-II expression induced by RAB22A^Q64L^ (Fig. 6b), subsequently impairing the packaging of activated STING into Refeesomes (Fig. 6c). Furthermore, knockdown of PI4K2A, but not PI4K2B, PI4KCA or PI4KCB, impeded the increase in LC3-II expression induced by RAB22A^Q64L^ (Supplementary information, Fig. S8a–d). Moreover, reintroduction of PI4K2A, but not its kinase-dead form PI4K2A^K152A^ could rescue the decrease of LC3-II in cells stably expressing RAB22A^Q64L^ with PI4K2A knockdown (Fig. 6d). In support of this finding, RAB22A^WT^ and RAB22A^Q64L^ co-localized with PI4K2A, but not with PI4K2B, PI4KCA or PI4KCB, in Rafeesomes (Supplementary information, Fig. S8e–i), and PI4K2A was detected both inside Rafeesomes (white arrowheads) and on the surface of Rafeesomes (blue arrowheads) (Supplementary information, Fig. S8e, f), as PI4K2A was localized to ER (Fig. 6e, g) or early endosomes.^52^ In addition, co-localization of RAB22A with PI4K2A at their endogenous levels was observed in NCI-H1975 cells (Fig. 6f). RAB22A, PI4K2A, and LC3 were co-localized in Rafeesomes in HeLa cells stably expressing GFP-RAB22A^WT^ or GFP-RAB22A^Q64L^ (Supplementary information, Fig. S8j). Importantly, RAB22A, PI4K2A, and calnexin were co-localized in Rafeesomes in HeLa cells stably expressing GFP-RAB22A^WT^ or GFP-RAB22A^Q64L^ (Fig. 6g). Knockdown of PI4K2A inhibited the packaging of both calnexin and LC3 into Rafeesomes (Supplementary information, Fig. S8k, l). Interaction of RAB22A with PI4K2A was detected at their endogenous levels in cells (Fig. 6h). Collectively, these results demonstrate that PI4P generated by PI4K2A is necessary for RAB22A-mediated non-canonical autophagosome formation.

Next, we sought to investigate whether PI4K2A affects the secretion of activated STING via R-EVs. As shown in Fig. 6i, j, knockdown of PI4K2A inhibited the secretion of activated STING, but did not affect the intracellular STING signaling in 4T1 cells. Furthermore, knockdown of PI4K2A did not affect 4T1 cell viability in vitro or 4T1 tumor growth in an orthotopic mouse model, but impaired the antitumor effect of diABZI on 4T1 tumors (Fig. 6k–n). These results demonstrate that knocking down PI4K2A attenuates the antitumor effect of diABZI by inhibiting the secretion of activated STING via R-EVs.

### PI4P directly recruits Atg16L1 to the ER-derived membrane, thereby promoting non-canonical autophagy

**Fig. 7:**
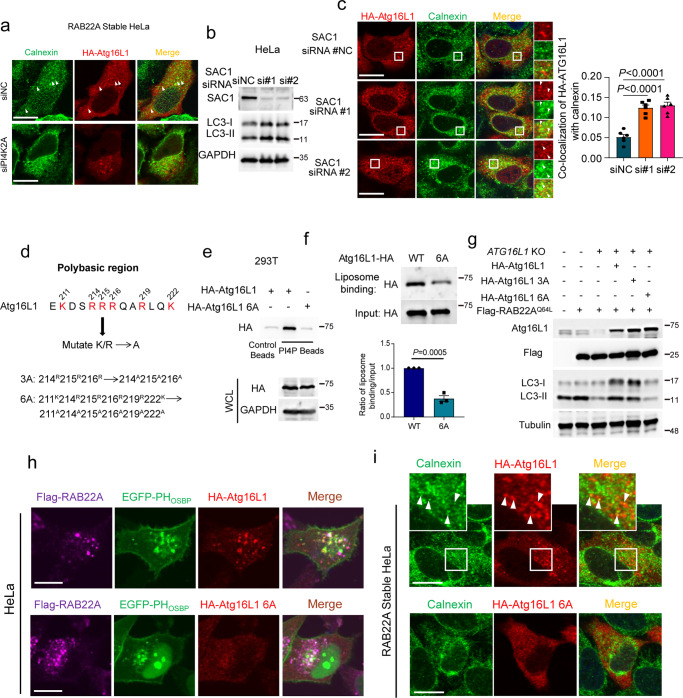
PI4P directly recruits Atg16L1 to the ER-derived membrane, thereby promoting non-canonical autophagy. **a** Immunofluorescence analysis of calnexin (green) and HA-Atg16L1 (red) in the RAB22A stable HeLa cells transiently expressing HA-Atg16L1. White arrowheads denote co-localization. Scale bar, 10 μm. **b** Western blot analysis of the WCL of the HeLa cells transiently expressing siRNAs targeting SAC1 for 48 h. **c** Immunofluorescence analysis of HA-Atg16L1 (red) and calnexin (green) in HeLa cells transiently expressing siRNAs targeting SAC1 for 48 h. Co-localization of HA-Atg16L1 with calnexin was quantified on the right. White arrowheads denote co-localization. *P* value was calculated by Student’s *t*-test. *n* = 6 fields. Scale bar, 10 μm. **d** Atg16L1 has a polybasic region. **e** Pull-down assay with PI4P-coated beads from 293T cells expressing WT HA-Atg16L1 and HA-Atg16L1 6A as indicated. **f** Liposome-binding assay showing that PI4P liposome has a higher binding affinity to HA-Atg16L1 WT proteins compared to HA-Atg16L1 6A mutant purified from HEK-293T cells. Ratio of liposome-binding proteins/input was quantified. *P* value was calculated by Student’s *t*-test. **g** Western blot analysis of WCL from stable Flag-RAB22A^Q64L^ HeLa cells with *ATG16L1* knockout and transient expression of HA-Atg16L1 WT or mutants of polybasic region-mutated HA-Atg16L1. **h** Immunofluorescence analysis of Flag (magenta), EGFP-PH_OSBP_ (green), and HA-Atg16L1/HA-Atg16L1 6A (red) in stable Flag-RAB22A HeLa cells transiently transfected with EGFP-PH_OSBP_ and HA-Atg16L1 or HA-Atg16L1 6A. Scale bar, 10 μm. **i** Immunofluorescence analysis of calnexin (green) and HA-Atg16L1 or HA-Atg16L1 6A (red) in stable Flag-RAB22A HeLa cells transiently transfected with HA-Atg16L1 or HA-Atg16L1 6A. White arrowheads denote co-localization. Scale bar, 10 μm.

PI3P binds to WIPI2 which subsequently recruits the Atg12–Atg5–Atg16L1 complex to generate LC3-II on phagophore.^46–48^ Atg16L1 plays key roles in localizing this complex to various membrane structures, where the complex catalyzes LC3 lipidation to promote phagophore formation.^45,53–55^ PI4P, but not PI3P or WIPI2, is required for the RAB22A-mediated non-canonical autophagosome formation, as shown above (Fig. 6a–c and Supplementary information, Fig. S7c, g–k). Notably, co-localization of Atg16L1 with calnexin was decreased in cells with PI4K2A knockdown (Fig. 7a), indicating that the recruitment of Atg16L1 to ER-derived membrane depends on PI4K2A. SAC1 is the PI4P phosphatase that locates on ER;^56,57^ therefore we asked whether knockdown of SAC1 would enrich PI4P leading to the recruitment of Atg16L1 onto ER-derived membrane and a subsequent increase of LC3-II. Indeed, we observed that Atg16L1 was co-localized with calnexin and that LC3-II was increased upon SAC1 knockdown in HeLa cells (Fig. 7b, c). Then, we sought to investigate how PI4P recruited Atg16L1 onto the ER-derived membrane. Atg16L1 has a polybasic region (aa 211–230) enriched in arginine (Arg) and lysine (Lys), which is positively charged and may directly bind to negatively charged phosphoinositide. Therefore, Atg16L1 6A was constructed, in which the six Arg/Lys residues within the polybasic region of Atg16L1 were mutated to alanine (Ala) (Fig. 7d). As expected, a pull-down assay using PI4P-coated beads showed that the binding ability of Atg16L1 6A to PI4P was markedly reduced compared with Atg16L1 WT (Fig. 7e). Furthermore, the liposomes made of synthetic lipids composed of phosphatidylcholine, phosphatidylethanolamine, and PI4P showed much stronger binding affinity to the purified Atg16L1 WT than to Atg16L1 6A (Fig. 7f). More importantly, Atg16L1 WT, but not Atg16L1 6A, rescued the decrease in LC3-II in cells stably expressing RAB22A^Q64L^ with *ATG16L1* knockout (Fig. 7g). Moreover, by using EGFP-PH_OSBP_ that specifically binds PI4P, we observed that Atg16L1, but not Atg16L1 6A, co-localized with both EGFP-PH_OSBP_ and calnexin in HeLa cells stably expressing Flag-RAB22A (Fig. 7h, i). Taken together, these results indicate that RAB22A engages PI4K2A to generate PI4P, which acts as the effector phosphoinositide on the ER-derived membrane and recruits the Atg12–Atg5–Atg16L1 complex, initiating the formation of non-canonical autophagosome.

## Discussion

**Fig. 8:**
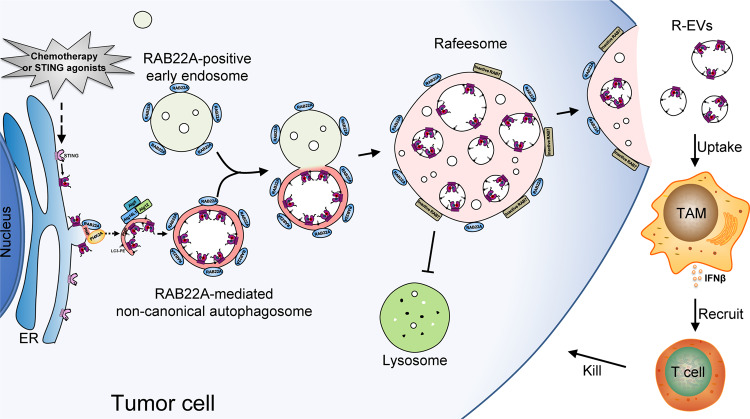
The proposed model for the intercellular transfer of activated STING conferring antitumor immunity. In tumor cells, RAB22A engages PI4K2A to generate PI4P, which recruits the Atg12–Atg5–Atg16L1 complex to facilitate LC3 lipidation on the ER-derived membrane. This promotes the formation of non-canonical autophagosomes, in which activated STING stimulated by agonists or chemoradiotherapy is localized. These RAB22A-induced autophagosomes fuse with RAB22A-positive early endosomes to become Rafeesomes. In addition, RAB22A inactivates RAB7, suppressing the fusion of Rafeesomes with lysosomes. This enables the inner vesicles of non-canonical autophagosomes bearing activated STING within Rafeesomes to be secreted as R-EVs, which activate the IFNβ pathway in recipient cells in the tumor microenvironment, enhancing antitumor immunity.

In tumor cells, as illustrated in Fig. 8, RAB22A binds to PI4K2A that generates PI4P, which recruits the Atg12–Atg5–Atg16L1 complex to induce LC3-II anchoring on the ER-derived membrane to form the RAB22A-mediated non-canonical autophagosome. Activated STING stimulated by agonists or chemoradiotherapy is localized on the membrane of such an autophagosome. This non-canonical autophagosome fuses with RAB22A-positive early endosome to become Rafeesome. In addition, RAB22A inactivates RAB7 to suppress the fusion of Rafeesome with lysosome, and the inner vesicles bearing activated STING are secreted as R-EVs, which activate the IFNβ pathway in recipient cells in the tumor microenvironment to execute antitumor immunity.

RAB GTPases locate on the surface of specific membranes and regulate vesicle formation, transport, and fusion through the recruitment of specific effector proteins.^37–39^ Numerous RAB proteins, including RAB1, RAB5, RAB7, RAB9A, RAB11, RAB23, RAB32, RAB33B, etc., have been shown to be involved in various stages of autophagosome formation.^58^ For instance, RAB5A has been reported to be involved in the formation of autophagosomes by regulating the autophagy-scavenging of toxic mutant Huntington proteins.^59^ RAB5A is a component of the autophagy initiation complex, which can activate VPS34 to generate PI3P that is involved in autophagosome formation.^58^ Then, RAB5 recruits the guanine nucleotide exchange factor of RAB7, the MON1–CCZ1 complex, to activate RAB7 mediating the fusion of autophagosomes with lysosomes.^60^ Therefore, RAB5-regulated autophagy may mainly be responsible for the degradation of cargoes. Most interestingly, as described in this report, PI4P generated by PI4K2A is identified as a key phosphoinositide required for the formation of RAB22A-mediated non-canonical autophagosomes, whereas PI3P acts as the phosphoinositide necessary for the formation of conventional autophagosomes.^46,47^ This RAB22A-mediated non-canonical autophagosome is destined to Rafeesome, through which the inner vesicle of the autophagosome originated from ER is finally secreted as R-EV. This process is different from not only the ER-phagy that is involved in the degradation of ER,^61,62^ but also the secretory autophagy which is responsible for the secretion of the cytosolic cargo.

RAB5A has also been shown to be involved in the formation of endocytic vesicles and their fusion with early endosomes. RAB5 recruits the MON1–CCZ1 complex to activate RAB7, and this promotes the conversion of early endosomes into late endosomes.^63,64^ Then, RAB7 mediates the transport of late endosomes along microtubules and the fusion of late endosomes with lysosomes, enabling the degradation of cell membrane proteins through the endolysosomal network.^65^ Although both RAB31 and RAB22A belong to the RAB5 subfamily, they have different functions in vesicular transport. We have recently reported that RAB31 engages FLOTs to drive ILV formation and inactivates RAB7 to suppress the fusion of MVEs with lysosomes, enabling the secretion of ESCRT-independent exosomes.^18^ In the current study, we found that the RAB22A-mediated non-canonical autophagosome fuses with RAB22A-positive early endosome and is transformed into Rafeesome, a novel organelle; and RAB22A inactivates RAB7 to suppress the fusion of Rafeesome with lysosome, enabling the secretion of the inner vesicle of autophagosome as R-EV.

In essence, exosomes are ILVs formed by the inward budding of endosomal membrane during the maturation of endosomes into MVEs, which are intermediates within the endosomal system, and secreted upon fusion of MVEs with the plasma membrane.^14^ Because the endosome is a monolayer membrane structure, ILVs can only be formed by budding inwards. However, autophagosomes are double-membrane structures and can fuse with endosomes to persist in the inner vesicles because of the topological principle of membrane structure. It has been reported that the fusion of autophagosomes with late endosomes is followed by degradation of the inner layer of the autophagosomes to form LC3-positive bullae, amphisomes.^66,67^ In this report, the formation of Rafeesome by fusion of RAB22A-mediated non-canonical autophagosome with early endosome is followed by secretion of the inner vesicles of autophagosomes as R-EVs. We speculate that the different fates of amphisome and Rafeesome may be a result of the difference in acidity between early and late endosomes.

Regarding to how the LC3-associated ILVs are formed, Leidal et al. reported that the LC3-conjugation machinery is required for packaging and secreting diverse RNA-binding proteins (RBPs) and small non-coding RNAs,^68^ showing that RBPs enter RAB5-positive MVBs, as co-localization of LC3 and CD63 in these MVBs was observed by confocal microscopy. They found that knockdown of either nSMase2 or FAN inhibited the entry of LC3 into MVBs and the reduction of RBP secretion. This may be insufficient to conclude that LC3 enters MVBs through budding,^68^ as nSMase2 was reported to mediate starvation-induced autophagy, and LC3 puncta formation was reduced by the nSMase2 inhibitor GW4869.^69^ In addition, Barman et al. revealed that nSMase2 is highly associated with the ER but rarely associated with MVBs, suggesting that the ER-located nSMase2 may be required for LC3 lipidation, providing a model whereby the transfer of ceramide from the ER mediates vesicle formation and is coupled to the selection of ER-associated RNA–RBP cargoes.^70^ Therefore, it is possible that inhibition of nSMase2 or FAN may block the formation of autophagosome rather than the entry of LC3 into MVBs. Collectively, these results suggest that the entry of LC3 into MVBs is unlikely through budding. Moreover, as shown in Supplementary information, Fig. S4a, active STING and CD63 co-localized in Rafeesomes, however, SDCBP knockdown inhibited the entry of CD63, but not active STING, into Rafeesomes. Notably, the electron microscopy result, as shown in Fig. 4e, revealed that there were two types of ILVs in Rafeesomes, which cannot be distinguished by confocal microscopy, and we speculate that the LC3-related ILVs via fusion were larger ones whereas the CD63-related ILVs via budding were smaller ones. The fusion of autophagosomes with early endosomes generates Rafeesomes, while the fusion of autophagosomes with late endosomes forms amphisomes.^66,67^

In this report, we reveal that R-EVs represent a novel type of EV that contain activated STING to execute antitumor immunity. Upon cGAMP stimulation, STING is activated and released from ER, we found that more activated STING was gradually co-localized with calnexin in NCI-H1975 cells compared to those with depletion of *RAB22A* (data not shown), indicating that activated STING upon cGAMP stimulation may be partially captured by the RAB22A-mediated autophagosome derived from ER before its translocation to Golgi and late endosome. The RAB22A level in tumor tissues and the STING level in serum may predict the response of cancer patients to chemoradiotherapy (Figs. 2i and 3n), and RAB22A renders tumor cells more sensitive to diABZI in mice by secreting more activated STING via R-EVs (Fig. 3j–l). Notably, similar to its active form RAB22A^Q64L^, some RAB22A mutants detected in human cancers, such as D31H, D31Y, P32S, M43I, V84L, S94I, R104Q, and E127Q, were more effective in increasing LC3-II in cells and inducing Refeesomes containing active STING compared to RAB22A^WT^ (Supplementary information, Fig. S9a, b), and these mutants also promoted STING^V155M^ secretion via EVs at the similar level compared with RAB22A^Q64L^ (Supplementary information, Fig. S9c), suggesting that cancer patients harboring these RAB22A mutants may benefit more from chemoradiotherapy. We are investigating the mechanism underlying the formation of RAB22A-mediated non-canonical autophagosomes in ER, and have already obtained some interesting findings. One is that the packaging of ER proteins into RAB22A-mediated non-canonical autophagosomes is selective. For instance, some ER proteins, such as calnexin and TRAM1, but not ATL3, Climp63, or inactive STING, can be packaged into this type of autophagosome. The mechanism of how active STING is packaged into the RAB22A-mediated non-canonical autophagosome is under investigation. Collectively, this study highlights the great potential of R-EVs containing activated STING as a new strategy for antitumor immunotherapy.

In addition, it has been recently reported that TBK1 is inhibited in tumor cells bearing p53 mutants,^71^ suggesting that p53 mutants may impair the intracellular STING signaling pathway; however, intercellular STING signal transduction via R-EVs is unlikely to be affected, as the activated STING in EVPs derived from p53-mutant MDA-MB-231 cells treated with diABZI was functional (Supplementary information, Fig. S1g). Most interestingly, STING has been detected in EVs derived from HSV-1-infected cancer cells, which execute antiviral activity in recipient cells.^72,73^ STING secretion discovered in these studies may also employ the R-EV pathway described in this report.

In summary, we define a new organelle, Rafeesome, through which the inner vesicle of RAB22A-mediated non-canonical autophagosome bearing activated STING is transferred and finally secreted as R-EV, a novel EV that triggers antitumor immunity in recipient cells. Our work, for the first time, provides evidence that Rafeesome regulates the intercellular transfer of the ER protein STING and that the inner vesicle of autophagosome originated from ER is secreted as a new type of EV (R-EV), proposing a new perspective for understanding the intercellular communication of organelle membrane proteins.

## Materials and methods

### Cells

NCI-H1975, HeLa, HCT116, B16F10, SUNE1, 4T1, THP-1, MDA-MB-231, L929, CT26, HL-60, Jurkat, and HEK-293T cell lines were obtained from ATCC. *STING*^−/−^ HeLa cells were gifts from Dr. Zhengfan Jiang (Peking University),^74^ and *ATG5*^−/−^ HeLa cells were gifts from Dr. Feng Shao (Beijing Institute of Biological Science).^54^ *STING*^−/−^ NCI-H1975 is a monoclonal cell line generated in our laboratory. NCI-H1975, B16F10, 4T1, MDA-MB-231, L929, CT26, HEK-293T, HeLa, and derived cells were cultured in DMEM (Gibco). HL-60, Jurkat, SUNE1, and THP-1 cells were cultured in RPMI 1640 medium (Gibco). HCT116 cells were cultured in McCoy’s 5A medium (Gibco) with 10% FBS (ExCell Bio) and 100 U/mL penicillin-streptomycin and were maintained in a humidified, 5% CO_2_ atmosphere at 37 °C. All cell lines used in this study were authenticated using short-tandem repeat profiling less than 6 months before the project was initiated and were not cultured for more than 1 month.

Mouse BMDMs were generated from Dr. Xiaojun Xia’s laboratory (Sun Yat-sen University Cancer Center (SYSUCC)). BMDMs were isolated from C57BL/6 mouse femurs and maintained in L929 conditioned medium (DMEM supplemented with 10% FBS, 10% L929 supernatant, and 100 U/mL penicillin-streptomycin) for 5 days. The cells in suspension were discarded, and adherent BMDMs were used in the following experiments.

### Reagents

Cisplatin (S1166), Adriamycin (S1208), Hydroxyurea (S1896), Rucaparib (S1098), 5-Fluorouracil (S1209), DMXAA (S1537), BFA (S7046), Rapamycin (S1039), and 3-MA (S2767) were from Selleck. Doxycycline hyclate (D9891), Phenylarsine oxide (P3075), GTP-Agarose suspension (G9768), Protein A agarose (P3476), mouse monoclonal Anti-Flag M2 Affinity Gel (A2220), and mouse monoclonal Anti-HA-Agarose antibody (A2095) were from Sigma-Aldrich. DiABZI STING agonist-1 (Tautomerism) (HY-112921) and Digitonin (HY-N4000) were from MCE. HaloTag® Ligands (GA1110) for super-resolution microscopy were from Promega. 2'3'-cGAMP (tlrl-nacga23-1) was from Invivogen. Pierce™ Anti-DYKDDDDK Magnetic Agarose (A36797) was from Thermo Fisher Scientific.

### Antibodies

Anti-TIM23 (NBP180680) was from Novus Biologicals. Anti-CD63 (ab134045), CD3 (ab16669), Atg7 (ab133528), STING (ab181125) and RAB22A (ab137093) were from Abcam. Anti-FLOT2 (3436), CD9 (13403), β-tubulin (2128), β-actin (4970), V5-Tag (13202), Histone H3 (4499), Flag (14793), Flag (8146), Fibronectin (26836), EEA1 (3288), cGAS (15102), HA (3724), HA (2367), Atg5 (12994), STING (13647), phospho-STING (Ser366) (19781), TBK1 (3504T), phospho-TBK1 (Ser172) (5483), phospho-IRF-3 (Ser396) (4947), CD8 (98941) and Atg16L1 (8089T) were from Cell Signaling Technology. Anti-FIP200 (GTX129093), WIPI2 (GTX132453) and PI4KCA (GTX107441) were from GeneTex. Anti-GM130 (610822) was from BD Biosciences. STING (19851-1-AP), Syntenin-1 (22399-1-AP), PI4KCB (13247-1-AP), p62 (18420-1-AP), IRF3 (11312-1-AP), GAPDH (10494-1-AP), Alix (12422-1-AP), SAC1 (13033-1-AP), and rabbit IgG (30000-0-AP) were from Proteintech Group. Anti-CD81 (sc-166029), TBC1D2B (sc-398906), PI4K2A (sc-390026), HSP70 (sc-24), calnexin (sc-11397), CD63 (sc-5275), and mouse IgG (sc-2025) were from Santa Cruz Biotechnology. Anti-STING (MA5-26030) was from Thermo Fisher Scientific. Anti-Tsg101 (HPA006161), RAB22A (HPA066920), LC3B (L7543), and IgG conjugated to 10-nm gold particles (G7777, G7402) were from Sigma-Aldrich. Secondary Antibody Alexa Fluor-594 (A-11037, A-11037), -568 (A-11036), -647 (A-21236), -488 (A-11034, A32723) were from Invitrogen. HRP-conjugated antibody (W4011, W4021) was from Promega.

### Plasmids

The constitutively active RAB GTPase library, FLOT1-HA, FLOT2-HA, and SBP-RILP constructs were generated as previously described.^18^ GFP-LC3, GFP-RFP-LC3 constructs were gifts from Dr. Min Li (Sun Yat-sen University),^75,76^ HaloTag plasmids were gifts from Dr. Dong Li (Chinese Academy of Sciences),^77^ and the EGFP-PH_OSBP_ construct was a gift from Shunji Jia (Tsinghua University).^78^ The cDNAs of *RAB22A* and *STING* were obtained by PCR and cloned into a pSIN vector with or without a tag (HA, Flag, V5, GFP, Halo, or mCherry). STING^V155M^, STING^V155M/LIR467^, STING^R281Q^, and Atg16L1 (with K/R changed to A) constructs were generated by PCR using forward primers containing mutated sites step by step. L-Flag-STING^V155M^-GFP was cloned by PCR from STING^V155M^-GFP with a Flag-coding sequence inserted between aa 112 and aa 113. Truncation mutants of Atg16L1 (aa 211–230) were generated by PCR from WT Atg16L1. The fusion cassettes of GFP-FKBP-INPP5E, GFP-FKBP-SAC1, FRB-Flag-RAB22A^Q64L^, and 3× Flag-Atg16L1(211–230)-GFP were generated by PCR with a One Step cloning kit (Vazyme). Tet-on GFP-RAB22A^Q64L^ was obtained by PCR and cloned into a pSIN vector with a tet-on transactivator. The shRNA expression constructs were inserted into a pLKO.1-puro backbone. Tet-on shRNA targeting *RAB22A* was cloned into a pLKO.1-puro backbone with a tet-on transactivator. sgRNA sequences were designed with the web application GUIDES (http://guides.sanjanalab.org/)^79^ and inserted into lentiCRISPRv2 plasmids. Sequences of sgRNA used in this study are shown in Supplementary information, Table S1.

All the mentioned constructs were fully verified by Sanger sequencing.

### Transfection, lentivirus, and stable cell line construction

For transient transfection, cells were transfected with plasmids using polyethylenimine (PEI) (Polysciences) or Lipofectamine 3000. The culture medium was refreshed after 6 h. Cells were transfected with siRNAs (Ribobio) using RNAiMAX (Thermo Fisher), and the culture medium was refreshed after 6 h. The mRNA level of the target gene was quantified 72 h after transfection.

Lentiviral production for gene overexpression, sgRNA, and Cas9 expression was performed as follows. HEK-293T cells were co-transfected with 3 μg of lentiCRISPRv2-sgRNA/pSin-EF2-cDNA, 2 μg of psPAX2 (gag, pol), and 1 μg of pMD2G using 24 μL of PEI (2 mg/mL) after being seeded for 24 h. Viral supernatants were collected 48 h after transfection and filtered through 0.45-μm PVDF filters (Millipore). Cells in six-well plates were infected with a virus at appropriate viral titers in the presence of 10 μg/mL polybrene (Sigma) and centrifuged at 2000 rpm (800× *g*) for 60 min at 37 °C. Stable cell lines were selected using 0.5 μg/mL puromycin.

### EVP isolation

EVP-free FBS was prepared by overnight ultracentrifugation at 100,000× *g*. Cells were washed twice with PBS and cultured in DMEM supplemented with 10% EVP-free FBS for 48 h. The supernatants of the cultured cells were collected and subjected to sequential centrifugation steps (600× *g* for 10 min and 2000× *g* for 30 min) at 4 °C to eliminate cells and cellular debris. Next, the supernatants were transferred to new tubes and centrifuged at 100,000× *g* for 2 h to precipitate the EVPs. All pellets were washed in PBS and recentrifuged at the same speed before being resuspended in sterile PBS. The concentrations of EVPs were quantitated by BCA assay, and the EVPs were stored at –80 °C.

### Animal experiments

Animal care and experiments were performed in strict accordance with the Guide for the Care and Use of Laboratory Animals and the Principles for the Utilization and Care of Vertebrate Animals and were approved by the Animal Research Committee of SYSUCC (Approval no. 19120D). Female BALB/c mice were purchased from Beijing Vital River Laboratory Animal Technology Co., Ltd. 4T1 cells (5 × 10^5^) were subcutaneously injected into the right inguinal area of BALB/c mice (*n* = 6, aged 6 weeks). Six days post implantation, the mice were randomly assigned into five treatment groups: the PBS, –diABZI EVP, +diABZI EVP, *STING*^−/−^–diABZI EVP, and *STING*^−/−^+diABZI EVP groups. Mice were administered 200 μL of PBS, 5 µg of EVPs derived from WT or *STING*^−/−^ 4T1 cells treated with or without 10 µM diABZI three times a week via tail vein injection. Tumor sizes were measured by calipers three times a week. Tumor volume was calculated using the formula length × width^2^/2 (mm^3^). Mice were sacrificed 19 days after implantation, and then, the tumors were dissected and evaluated.

Vector, RAB22A, shNC, or shRNA-PI4K2A stable 4T1 cells (5 × 10^5^) were injected into the mammary fat pads through surgery in BALB/c mice. Six days post implantation, mice were treated with or without 1.5 mg/kg (vector vs RAB22A) or 3.0 mg/kg (shNC vs shRNA-PI4K2A) of diABZI three times per week by tail intravenous injection, as indicated, for another 14 days. Tumor size was measured using calipers every other day. Tumor volume was calculated using the formula length × width^2^/2 (mm^3^). The mice were sacrificed 20 days after implantation, and then, the tumors were dissected and evaluated.

### Fluorescence-activated cell sorting

For analysis of cytotoxic T cell infiltration in mouse tumors, mouse tumors were digested at 37 °C at 100 rpm for 1 h in digestion buffer (RPMI 1640 medium containing 0.4 mg/mL Collagenase IV (Sigma-Aldrich) and 50 U/mL DNase I (Sigma-Aldrich). The digested cell suspension was filtered by a 70-μm cell strainer to obtain single cell suspension and washed with staining buffer (PBS containing 2% FBS) twice and PBS once. Lymphocytes were obtained from peripheral blood lymphocyte isolates (TBD, LTS1077). Then cells were stained with Zombie Aqua™ Fixable Viability Kit (bv510) (Biolegend, 423101) in PBS for 15 min to stain the non-viable cells. After that, cells were washed with staining buffer twice and stained with APC anti-CD45 (Biolegend, 103111), FITC anti-CD3ε (Biolegend, 100203), and PE/cy7 anti-CD8α (Biolegend, 100721) antibodies at 4 °C for 30 min. Flow cytometry analysis was performed after cells were washed twice with staining buffer.

### Immunoblot and immunoprecipitation

For western blotting, the cells were washed once with cold PBS and then lysed on ice in RIPA buffer (50 mM Tris-HCl, pH 7.5, 150 mM NaCl, 1 mM EDTA, and 1% NP40) supplemented with Protease Inhibitor Cocktail Set I (Calbiochem; 539131) and Phosphatase Inhibitor Cocktail Set II (Calbiochem; 524625). The lysates were cleared by centrifugation at 12,000 rpm for 20 min at 4 °C. For immunoprecipitation assay, the antibody beads were washed three times with RIPA buffer. Subsequently, 20 μL of the beads were added and incubated with the lysates for 6 h at 4 °C. For endogenous immunoprecipitation assay, protein A or protein A/G agarose beads were washed three times with RIPA buffer, and then, an antibody or the control rabbit or mouse IgG was added to the cell lysates with the washed agarose beads, followed by incubation for 6 h at 4 °C. The beads were then washed five times with RIPA buffer. The immunoprecipitates and cell lysates were then boiled in gel loading buffer for 10 min and loaded onto sodium dodecyl sulfate-polyacrylamide gels. The proteins in the gels were transferred to Immobilon-P PVDF membranes (Millipore), which were then blocked in PBS with 5% nonfat milk and 0.1% Tween-20 and probed with primary antibodies overnight at 4 °C. Secondary HRP-conjugated antibodies were then added, and Clarity ECL substrate (Bio-Rad) or High-sig ECL substrate (Tanon) was used for detection by MiniChmei Chemiluminescence imager (SAGECREATION, Beijing).

### Immunohistochemistry and immunofluorescence

Freshly collected tumors were fixed with 4% paraformaldehyde and embedded in paraffin before they were sectioned into 4-μm thickness. Then, the slices were subjected to deparaffinization, antigen retrieval, and blocking. For Immunohistochemistry analysis, the slices were incubated with anti-RAB22A antibody for 2 h at room temperature followed by incubation with anti-rabbit IgG secondary antibody and DAB reagent. The sections were viewed under a microscope (EVOS FL Auto Cell Imaging System, USA).

For immunofluorescence staining, cells were fixed with 4% paraformaldehyde for 15 min, further permeabilized with 0.5% Triton X-100 (Sigma-Aldrich) for 15 min, and blocked with goat serum (ZSGB-BIO, ZLI-9056) for 30 min at room temperature. After blocking, the cells were stained with primary antibody for 2 h at room temperature or overnight at 4 °C and washed three times with PBS. Then, the cells were stained with a secondary antibody for 1 h followed by Hoechst 33342 (Invitrogen, H3570) for 2 min. After staining, the cells were washed three times with PBS. Then, the cells were mounted with an antifade mounting medium (Beyotime, P0128M) and imaged by a confocal microscope (Zeiss LSM880 with Airyscan). Structured illumination microscopy (SIM) super-resolution images were taken using a Nikon N-SIM system with a 100× oil immersion objective lens, 1.49 NA (Nikon). Images were captured using Nikon NIS-Elements and were reconstructed using slice reconstruction in NIS-elements.

### Live-cell imaging

Time-lapse images of autophagosomes/MVB-like structures fusing with early endosomes were taken by a Nikon Ti2 spinning disk microscope. HeLa cells stably expressing tet-on GFP-RAB22A^Q64L^ were seeded on a 35-mm glass-bottom dish. Twenty-four hours later, they were transfected with either calnexin-mCherry or STING^V155M^-Halo and treated with 50 ng/mL doxycycline for another 24 h. Before imaging, cells transiently expressing STING^V155M^-Halo were pre-incubated with 30 nM HaloTag Ligands for 30 min at 37 °C, and then the culture medium was refreshed. Time-lapse movies were recorded using images captured at a 10–20 s interval.

### Cell stimulation with 2’3’-cGAMP

Cells were treated with 2 µM 2’3’-cGAMP in digitonin permeabilization solution (50 mM HEPES, pH 7.0, 100 mM KCl, 3 mM MgCl_2_, 0.1 mM dithiothreitol, 85 mM sucrose, 0.2% BSA, 1 mM ATP, and 10 µg/mL digitonin) for 30 min at 37 °C. Cells were then incubated in a fresh medium for 1 h before western blot analysis or in an EVP-free medium for 48 h before EVP isolation.

### Establishment of a heterozygous STING^V155M/WT^ knock-in HeLa cell line

The establishment of endogenous heterozygous STING^V155M/WT^ knock-in HeLa cell line was processed by an improved method based on homology-directed repair, as indicated before.^80^ Briefly, an efficient sgRNA (5’-AUCGAGAAAUGGGGGCAGAG-3’) targeting *STING* intron 3 was cloned into pLenti-CRISPR-V2. A donor DNA template containing cytomegalovirus promoter-driven DsRed-expressing cassette flanked by the left and right homology arms (HAs) was used. The desired V155M mutation was located on the left arm. Because the donor template carried an independent expressing cassette, the expression of dsRed does not affect STING expression. Donor template was amplified by PCR and purified. Then the sgRNA-Cas9 and donor template were co-transfected into HeLa cells. Six days later, dsRed-positive cells were sorted by flow cytometry, and positive clones were selected. cDNA of these cells was analyzed by Sanger sequencing to identify clones that have been successfully edited.

### Quantitative reverse transcription (qRT)-PCR assays

mRNA was extracted with an RNA extraction kit (TIANGEN) according to the manufacturer’s instructions. cDNA for each sample was generated using 1 μg of total mRNA and RT SuperMix for qPCR (Vazyme). Then, quantitative PCR assays were performed using SYBR Color qPCR Master Mix (Vazyme) with a LightCycler 480 (Roche). For the details of the qRT-PCR primers used in this study, please see Supplementary information, Table S2.

### TEM

Cells with confluence at 80%–90% were centrifugated, then the cell pellet was pre-fixed using 2.5% neutral glutaraldehyde (Ala Aesar, A17876) diluted in 0.1 M phosphate buffer for 2 h at 4 °C. After that, the pellet was rinsed with 0.1 M phosphate buffer six times for 30 min each. Then the pellet was post-fixed with 1% osmic acid (TED PELLA, 18456) diluted in 0.1 M phosphate buffer for 1 h at 4 °C followed by washing with 0.1 M phosphate buffer three times for 5 min each. Pellets were then dehydrated in gradient ethanol (30%, 50%, 70%, 90%, and 100%) for 5 min each. Pellets were infiltrated with a 1:1, 1:2, and 0:1 mixture respectively of acetone and Epon812 resin (TED PELLA, GP18010) at 38 °C for 3–4 h, and then were embedded in Epon812 resin. After being polymerized at 37 °C, 45 °C, and 60 °C for 12 h, 12 h, and 48 h, respectively, the resin was ultra-thin sectioned using a 70 nm-thick ultramicrotome (UC-7). Then the sections were double stained with 2% uranyl acetate (EMS, 22400) for 30 min and lead citrate (TED PELLA, 19314) for 15 min. After being dried in the air, samples were observed with a transmission electron microscope (Japan Electron Optics Laboratory Co., Ltd., JEM-1400 PLUS) at a voltage of 100 kV. Images were captured using a CCD camera (EMSIS, MORADA).

### IEM

Cells were prepared for IEM with LR White resin (14381-UC, ELECTRON MICROSCOPY SCIENCES) as previously described.^18^ Briefly, cells were pelleted at 150× *g* for 8 min and fixed in a solution containing 2% paraformaldehyde, 0.05% glutaraldehyde, and 0.1 M PBS (pH 7.4) for 90 min at 4 °C. The fixed pellets were washed three times with 0.1 M PBS (pH 7.4) for 10 min each time at 4 °C and then dehydrated with a 30%, 50%, 70%, and 90% graded ethanol series at –20 °C; each ethanol dehydration step lasted for 20 min, and the 30% ethanol dehydration step was performed at 4 °C. Samples were infiltrated with 40%, 70%, and 100% LR white-ethanol series at –20 °C for 1 h per step, followed by infiltration with 100% LR white overnight at –20 °C. The resin-containing sample was then polymerized in a PCR tube by UV irradiation (360 nm) at –20 °C for 72 h and at room temperature for 48 h. Immunolabeling was performed with a rabbit anti-HA antibody (1:20; Cell Signaling; 3724) for 2 h at 37 °C, followed by incubation with goat anti-rabbit IgG conjugated to 10-nm gold particles (1:20; Sigma; G7402) as the secondary antibody for 2 h at 37 °C. The samples were visualized at an accelerating voltage of 120 kV with a JEOL JEM-1400 transmission electron microscope with an AMT XR41 digital imaging system.

### High-resolution (12%–36%) iodixanol density gradient fractionation

A 400-mL cultured supernatant sample of HeLa cells was subjected to ultrafiltration to obtain the concentrated extracellular matter. The concentrated extracellular matter was isolated by iodixanol density gradient fractionation as previously described.^67^ Briefly, iodixanol (OptiPrep) density media (Sigma-Aldrich, D1556) was prepared in ice-cold PBS immediately before use to generate discontinuous step (12%–36%) gradients. The concentrated extracellular matter was resuspended in ice-cold PBS and mixed with ice-cold iodixanol/PBS for a final 36% iodixanol solution. The suspension was added to the bottom of a centrifugation tube, and the solutions with descending concentrations of iodixanol in PBS were carefully layered on top, creating a complete gradient. The bottom-loaded 12%–36% gradient layer was subjected to ultracentrifugation at 120,000× *g* for 15 h at 4 °C with an SW41 TI rotor (Beckman Coulter). Twelve individual 1-mL fractions were collected from the top to bottom of the gradient. Each 200-μL fraction was transferred to new tubes, 50 μL of 5× loading buffer was added, and the tubes were boiled for 10 min. Each 20-μL sample was subjected to SDS-PAGE and western blotting.

### RAB22A^Q64L^-positive sub-organelle immunoprecipitation

HeLa cells stably expressing Flag-RAB22A or Flag-RAB22A^Q64L^ were seeded on a 15-cm dish for 24 h. Upon reaching 90% confluence, the cells were washed and scraped into ice-cold KPBS buffer (136 mM KCl and 10 mM KH_2_PO_4_ at pH 2.5 adjusted with KOH) containing protease inhibitor, and then, the cells were pelleted by centrifugation at 2000× *g* for 1 min at 4 °C. The pellets were resuspended in 200 μL of KPBS buffer, and 10 μL of this suspension was used as the whole-cell control. Then, the remaining cells were homogenized with 40 strokes in a 2-mL Dounce homogenizer (Sigma, D8938). Next, the homogenate was centrifuged at 3000× *g* for 3 min at 4 °C. Supernatants containing anti-DYKDDDDK beads (Thermo Scientific™, A36797) washed three times with KPBS were incubated on a rotary shaker for 30 min at 4 °C. The mixture was placed on a magnetic rack to remove the supernatants, and then, the beads were washed three times with 1 mL of KPBS buffer. Beads containing immunoprecipitated samples were added to 40 μL of lysis buffer and boiled at 100 °C for use in western blot analysis.

### The ELISA for STING

The serum of a patient was added to 0.5% NP40 for 1 h on ice to lyse the vesicles completely, and the level of STING was detected following the instructions of an ELISA kit (Cloud-Clone Corp, HEN011Hu). In brief, first, 100 μL of serum sample was added to a 96-well strip plate and incubated for 1 h at 37 °C. Then, the liquid in each well was removed, 100 μL of reagent A working solution was added, and the plate was incubated for 1 h at 37 °C. The solution was aspirated, and the well was washed with 350 μL of 1× wash solution using a multichannel pipette three times. Next, 100 μL of reagent B working solution was added and incubated for 30 min at 37 °C. The wash process was repeated five times as described above. Ninety microliter of substrate solution was added to each well and incubated for 10–20 min at 37 °C until the liquid sample turned blue. Then, 50 μL of stop solution was added and mixed on a shaker gently. A microplate reader was used to immediately measure the solution at 450 nm. The level of STING was analyzed based on a standard curve made on the basis of a standard solution.

### GTP-binding assay

Cells were harvested upon reaching confluency in a culture of 90% and were suspended in binding buffer (20 mM HEPES, pH 8, 150 nM NaCl, and 10 mM MgCl_2_) containing a cocktail of protease and phosphatase inhibitors, lysed using three freeze–thaw cycles, and then centrifuged at 12,000 rpm for 20 min. The supernatants were incubated with 100 μL of GTP-agarose suspension (Sigma-Aldrich, G9768) for 2 h with rotation at 4 °C. The beads were pelleted by centrifugation, washed three times in binding buffer, and suspended in 40 μL of SDS-PAGE sample buffer. The proteins were boiled and subjected to SDS-PAGE and western blotting.

### Lyso-Tracker Red probe

HeLa cells stably expressing GFP-RAB22A or GFP-RAB5A were plated on glass-bottom plates. After 24 h of growth to confluence, the medium was replaced with a medium containing 50 nM Lyso-Tracker Red (Beyotime Institute, C1046), and the cells were treated for 30 min with 5% CO_2_ at 37 °C. The Lyso-Tracker Red was removed, and a complete medium was added. Images were obtained using a fluorescence microscope.

### Inducible recruitment of phospholipid phosphatases

In brief, we established a HeLa cell line stably expressing FRB-Flag-RAB22A^Q64L^ that was split onto slides overnight and then transiently transfected with GFP-FKBP-INPP5E or GFP-FKBP-SAC1 with Lipofectamine 3000 in Opti-MEM medium. The medium was changed to a regular medium 8 h after transfection. After culturing overnight, the cells were treated with rapamycin (1 μM) for 80 min before immunostaining. Images were obtained using a fluorescence microscope.

### PI4P binding assay

Pull-down using PI4P-coated beads was performed using cell lysates of 293T cells transfected with HA-Atg16L or HA-Atg16L 6A plasmid, in accordance with the manufacturer’s instructions (Echelon, P-B004A-2).

### Liposome preparation

Liposome was prepared as described in published paper^81,82^ with some modifications. A total of 900 μg lipids (500 μg POPC, 102 μg cholesterol, 182 μg DPPE, 53 μg DOPS, and 63 μg PI4P) was dissolved in CH_3_Cl:CH_3_OH:1N HCl (2:1:0.01) mixture in a glass bottle. The solution was dried under pressurized N_2_ and then vacuumed for 40 min to completely remove the solvent. 300 μL HEPES butter (50 mM HEPES, 100 mM KCl, pH 7.5) was added to the vial. The vial was kept in liquid nitrogen to freeze the buffer and then transferred to room temperature water to melt the ice. The procedure was repeated until the solution became clear. The mixture was then extruded 18 times through an 800-nm polycarbonate membrane (Whatman) using an extruder.

### Liposome-binding assay

HEK-293T cells transiently transfected with HA-Atg16L or HA-Atg16L 6A mutant were lysed in RIPA buffer as described above and the proteins were immunoprecipitated by HA agaroses. Proteins were eluted by 300 μg/mL HA peptide in HEPES buffer. The same amount of the two proteins was incubated with 30 μg liposome at room temperature for 30 min on a rotating wheel. Liposome was pelleted by centrifugation at 50,000 rpm using TLA110 rotor (Beckman) and then washed twice with HEPES buffer. The final protein-binding liposome was lysed in 20 μL RIPA buffer, and the amount of binding protein was analyzed by western blotting.

### Clinical specimens and evaluation

A total of 100 paraffin-embedded NPC patient tissue samples were obtained from SYSUCC. These patients were pathologically diagnosed between January and December 2013 and received radiotherapy with or without chemotherapy. The overall survival time was calculated from the day of diagnosis to the end of the follow-up day in November 2019 or the date of death because of recurrence and/or metastasis. An anti-RAB22A rabbit antibody (Sigma, HPA066920) was used for immunostaining. The staining index (SI) was determined by multiplying the score obtained for the staining intensity with the score obtained for the positive area. The intensity score was defined as follows: 0, negative; 1, weak; 2, moderate; and 3, strong. Tissue sections were examined and scored separately by two independent investigators blinded to the clinic pathological data. X-tile software (V.3.6.1; Yale University, New Haven, Connecticut, USA) was used to generate the optimal cutoff values for determining high and low RAB22A density in the tumor areas and the relationship of this density to overall survival. Patients with a high SI (≥ 2) were assigned to the RAB22A high-expression group, and those with a low SI (0 and 1) were assigned to the RAB22A low-expression group. Survival analyses were performed using the Kaplan–Meier method, and differences were compared by log-rank test.

The NPC patient serum samples described in this paper were collected during the course of chemotherapy or radiotherapy between April and November 2020 in SYSUCC. Then, ELISA was performed to detect the level of STING in the patient serum samples. Approximately 4–6 weeks after chemotherapy or at the end of radiotherapy, treatment responses were assessed radiographically by two senior clinicians specializing in NPC using Response Evaluation Criteria in Solid Tumors guidelines (version 1.1).^83^ Then, we compared the STING levels in the serum of NPC patients between the two RAB22A expression groups using *t*-tests.

This retrospective study was approved by the Ethics Committee of SYSUCC (Approval no. B2021-096-01) in accordance with the Declaration of Helsinki.

### MTT assay

A 3-(4,5-dimethylthiazol-2-yl)-2,5-diphenyltetrazolium bromide (MTT) assay was used to measure cell viability. Briefly, 4T1 cells were seeded at a density of 800 cells per well in a 96-well microplate. The cells were incubated with MTT for 4 h, and the optical density was detected at 490 nm with the microplate reader once per day for 4 days.

### Statistical analysis

GraphPad Prism (version 8.3.0) software was used for all statistical analyses. The significance of the differences was assessed using the two-tailed Student’s *t*-test. The correlations between RAB22A expression and overall survival curves were assessed using Kaplan–Meier plots and compared with the log-rank test. Data are presented as means ± SEM. Differences were considered significant when *P* values were < 0.05.

## Supplementary information

Supplementary Figure S1

Supplementary Figure S2

Supplementary Figure S3

Supplementary Figure S4

Supplementary Figure S5

Supplementary Figure S6

Supplementary Figure S7

Supplementary Figure S8

Supplementary Figure S9

Supplementary Table S1

Supplementary Table S2

Supplementary Table S3

Supplementary video legend

Supplementary Video S1

Supplementary Video S2
