# Supplementary material for: Intercellular transfer of activated STING triggered by RAB22A-mediated non-canonical autophagy promotes antitumor immunity
Source: Cell Res. 2022 Oct 24;32(12):1086–104. doi: 10.1038/s41422-022-00731-w (PMC9715632; doi:10.1038/s41422-022-00731-w)
Supplement: Supplementary file 1 — Supplementary Figure S1 [file 41422_2022_731_MOESM1_ESM.pdf]

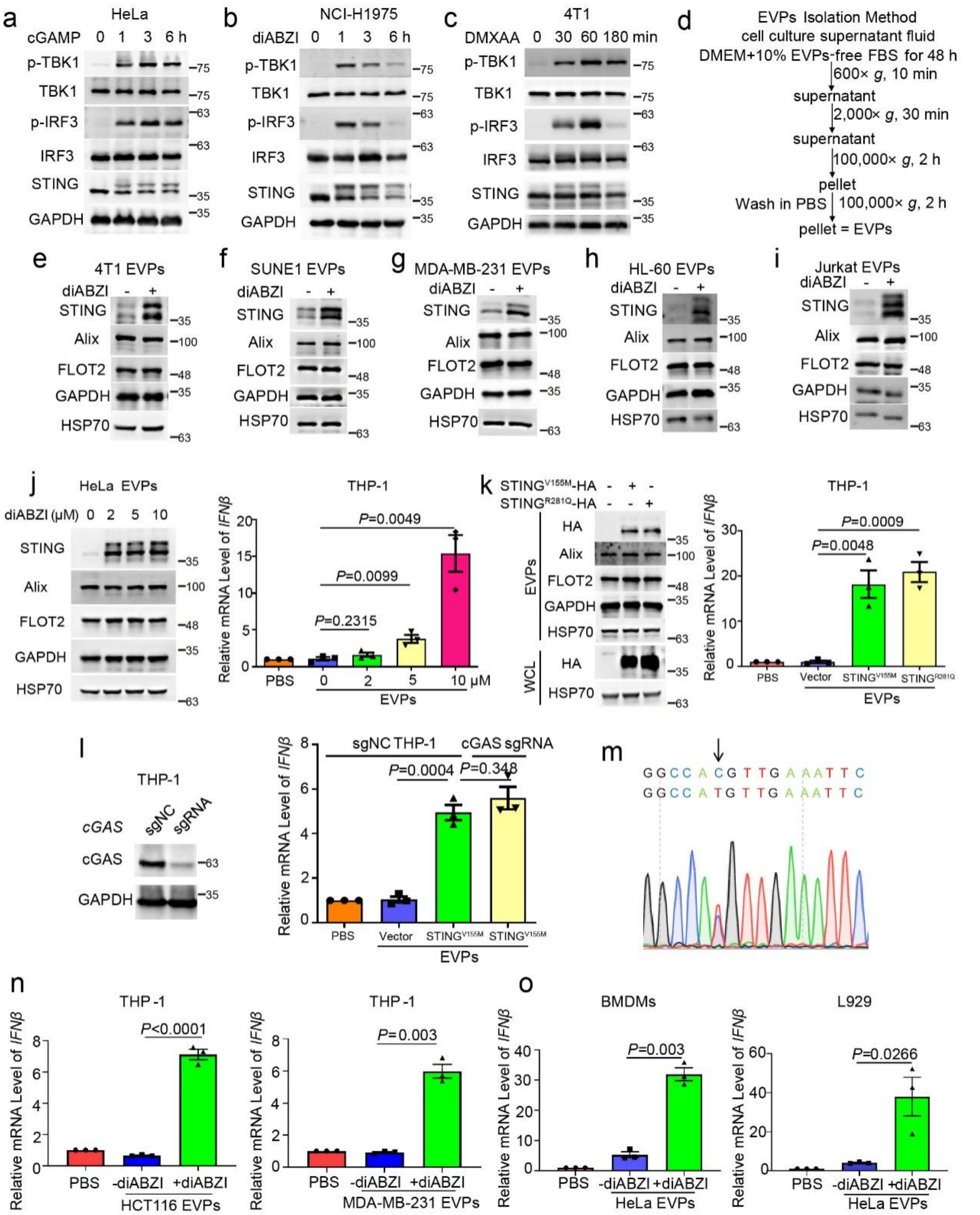

Supplementary information, Fig. S1 EVs containing activated STING can increase *IFNβ* in various recipient cells independent of cGAS.

a-c Western blot analyses of whole-cell lysates (WCL) from HeLa cells treated with1 μM cGAMP (**a**), NCI-H1975 cells treated with 10 μM diABZI (**b**), 4T1 cells treated with 50 μg/mL DMXAA (**c**) at the indicated times.

d The workflow of EVP isolation based on differential ultracentrifugation.

e-i Western blot analyses of the isolated EVPs derived from 4T1 (10 μM) (**e**),

SUNE1 (10 μM) (**f**), MDA-MB-231 (10 μM) (**g**), HL-60 (2 μM) (**h**) and Jurkat (5 μM) (**i**) cells treated with or without diABZI for 48 h.

j THP-1 cells were collected after exposure to EVPs for 24 h which derived from wild-type and $STING^{-/-}$ HeLa cells treated with (0, 2, 5, 10 μM) diABZI. The mRNA level of *IFNβ* was then quantified. *P* values were calculated by student's *t*-test.

k Whole-cell lysates and EVPs derived from HeLa cells stably expressing vector and activated forms of STING, as indicated, were prepared and subjected to Western blotting with the indicated antibodies. THP-1 cells were incubated with 2 μg/mL EVPs derived from the aforementioned stable HeLa cells for 24 h, and the mRNA level of *IFNβ* was quantified by qPCR. *P* values were calculated by student's *t*-test.

l Wild-type and $cGAS^{-/-}$ THP-1 cells were incubated with EVPs derived from HeLa cells stably expressing the vector and activated form of STING for 24 h, and the mRNA level of *IFNβ* was quantified by qPCR. *P* values were calculated by student's *t*-test.

m Sequencing analysis of the HeLa cells with $STING^{V155M/WT}$, the heterozygous mutant of $STING^{V155M}$.

n THP-1 cells were collected after exposure to EVPs for 24 h which derived from HCT116 or MDA-MB-231 cell lines treated with or without 10 μM diABZI. The mRNA level of *IFNβ* was then quantified. *P* values were calculated by student's *t*-test.

o BMDMs and L929 cells were collected after exposure to EVPs for 24 h which derived from HeLa cells treated with or without 10 μM diABZI. The mRNA level of *IFNβ* was then quantified. *P* values were calculated by student's *t*-test.
