# Supplementary material for: Intercellular transfer of activated STING triggered by RAB22A-mediated non-canonical autophagy promotes antitumor immunity
Source: Cell Res. 2022 Oct 24;32(12):1086–104. doi: 10.1038/s41422-022-00731-w (PMC9715632; doi:10.1038/s41422-022-00731-w)
Supplement: Supplementary file 2 — Supplementary Figure S2 [file 41422_2022_731_MOESM2_ESM.pdf]

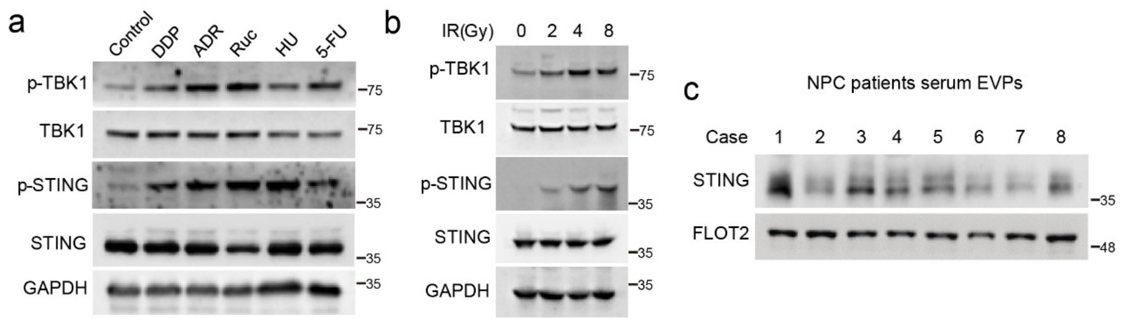

Supplementary information, Fig. S2 Chemotherapy drugs and ion radiation activated the STING signaling pathway.

a NCI-H1975 cells were treated with a variety of chemotherapy drugs (DDP: 3 μg/mL, ADR: 0.05 μg/mL, Ruc: 25 μM, HU: 0.6 mM, 5-FU: 50 μg/mL) for 48 h, and whole-cell lysates were subjected to Western blotting.

b NCI-H1975 cells were treated with different doses of ion radiation for 72 h, and whole-cell lysates were subjected to Western blotting.

c Western blot analyses of the serum EVPs from NPC patients after radiotherapy and/or chemotherapy.
