# Supplementary material for: Intercellular transfer of activated STING triggered by RAB22A-mediated non-canonical autophagy promotes antitumor immunity
Source: Cell Res. 2022 Oct 24;32(12):1086–104. doi: 10.1038/s41422-022-00731-w (PMC9715632; doi:10.1038/s41422-022-00731-w)
Supplement: Supplementary file 3 — Supplementary Figure S3 [file 41422_2022_731_MOESM3_ESM.pdf]

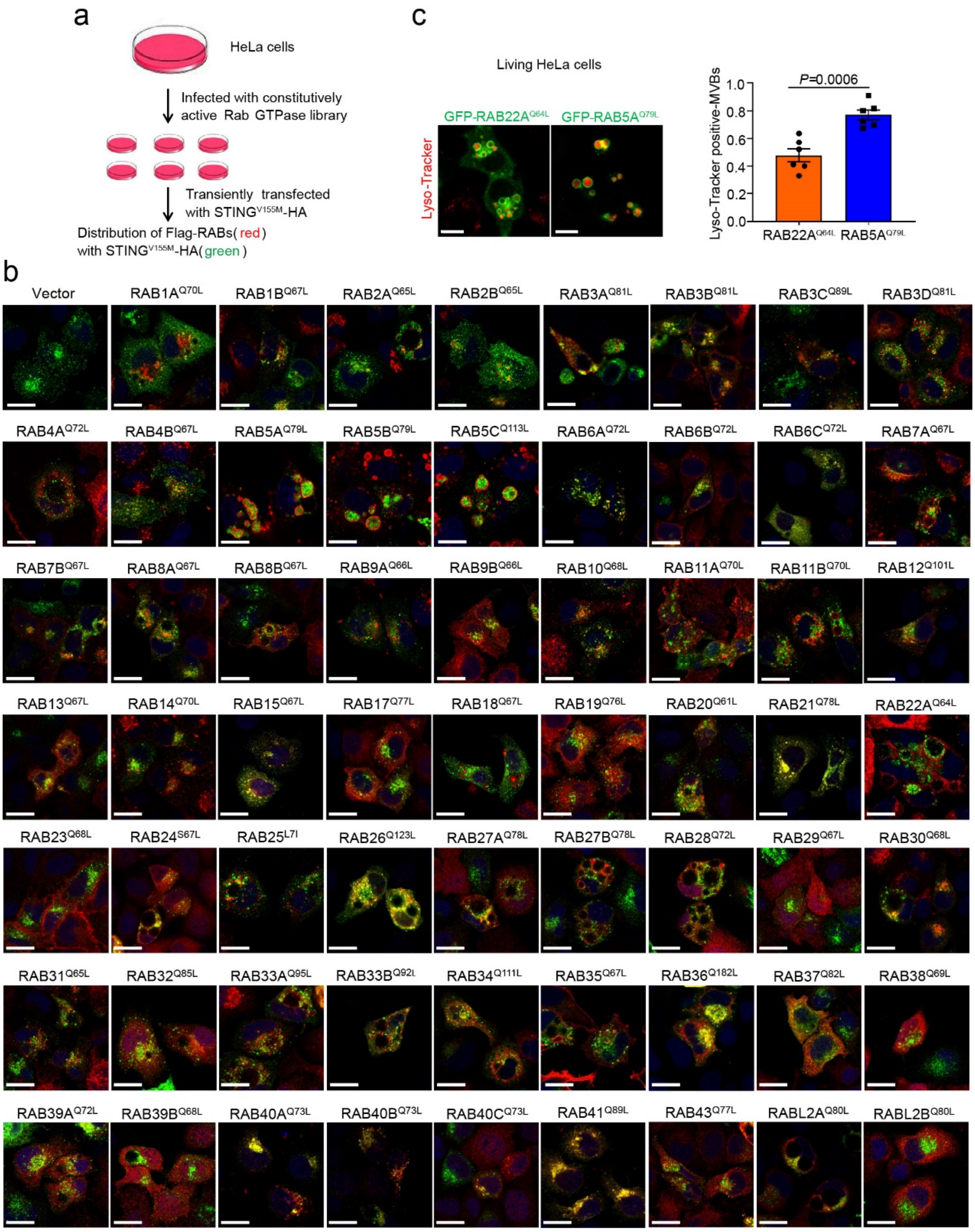
a
HeLa cells
Infected with constitutively active Rab GTPase library
Transiently transfected with STING^V155M-HA
Distribution of Flag-RABs(red) with STING^V155M-HA(green)
c
Living HeLa cells
GFP-RAB22A^Q64L
GFP-RAB5A^Q79L
Lyso-Tracker
Lyso-Tracker positive-MVBs
P=0.0006
1.0
0.8
0.6
0.4
0.2
0.0
RAB22A^Q64L
RAB5A^Q79L
b
Vector
RAB1A^Q70L
RAB1B^Q67L
RAB2A^Q65L
RAB2B^Q65L
RAB3A^Q81L
RAB3B^Q81L
RAB3C^Q89L
RAB3D^Q81L
RAB4A^Q72L
RAB4B^Q67L
RAB5A^Q79L
RAB5B^Q79L
RAB5C^Q113L
RAB6A^Q72L
RAB6B^Q72L
RAB6C^Q72L
RAB7A^Q67L
RAB7B^Q67L
RAB8A^Q67L
RAB8B^Q67L
RAB9A^Q66L
RAB9B^Q66L
RAB10^Q68L
RAB11A^Q70L
RAB11B^Q70L
RAB12^Q101L
RAB13^Q67L
RAB14^Q70L
RAB15^Q67L
RAB17^Q77L
RAB18^Q67L
RAB19^Q76L
RAB20^Q61L
RAB21^Q78L
RAB22A^Q64L
RAB23^Q68L
RAB24^S67L
RAB25^L71
RAB26^Q123L
RAB27A^Q78L
RAB27B^Q78L
RAB28^Q72L
RAB29^Q67L
RAB30^Q68L
RAB31^Q65L
RAB32^Q85L
RAB33A^Q95L
RAB33B^Q92L
RAB34^Q111L
RAB35^Q67L
RAB36^Q182L
RAB37^Q82L
RAB38^Q69L
RAB39A^Q72L
RAB39B^Q68L
RAB40A^Q73L
RAB40B^Q73L
RAB40C^Q73L
RAB41^Q89L
RAB43^Q77L
RABL2A^Q80L
RABL2B^Q80L

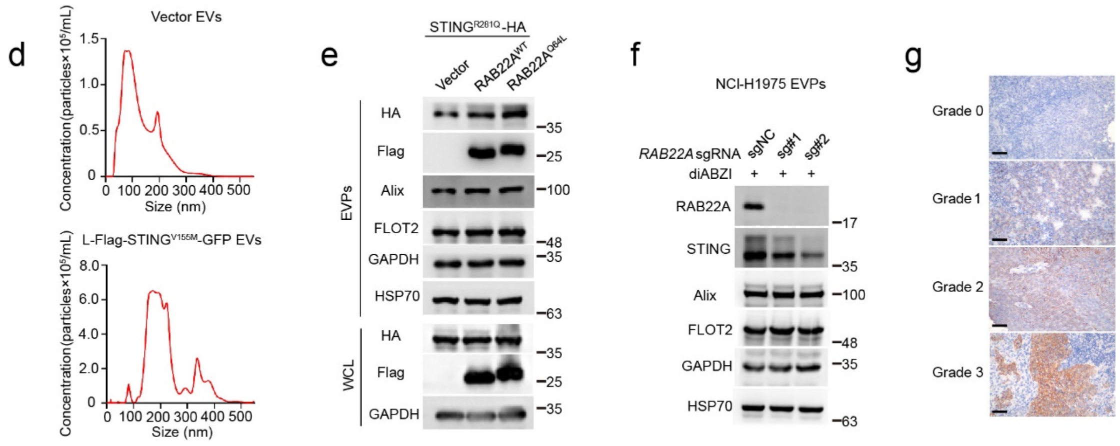

Supplementary information, Fig. S3 RAB22A controls the formation of activated STING-containing MVB-like structures and the secretion of activated STING-containing EVs.

a Schematic depicting the workflow of the screening method.

b Immunofluorescence analysis of STING^{V155M}-HA (green), Flag-Rabs (red) and DAPI (blue) in the indicated stable HeLa cells transiently expressing STING^{V155M}-HA. Scale bar, 10 μm.

c Immunofluorescence analysis of Lyso-Tracker (red) with GFP-RAB22A^{Q64L} or GFP-RAB5A^{Q79L} (green) in the indicated stable living HeLa cells. Lyso-Tracker positive-MVBs were quantified on the right. *n* = 6 fields. Scale bar, 10 μm.

d NanoSight nanoparticle tracking analysis of the EVPs isolated from the vector-stable HeLa cells and purified L-Flag-STING^{V155M}-GFP EVs.

e Western blot analyses of EVPs and lysates from the indicated stable HeLa cells simultaneously and stably expressing STING^{R281Q}-HA.

f Western blot analyses of EVPs derived from *RAB22A*-KO NCI-H1975 cells treated with diABZI.

g IHC staining of the NPC tumor tissue. Stained tumor tissue sections representative of grade 0, 1, 2, and 3 staining are shown. Scale bar, 50 μm.
