# Supplementary material for: Intercellular transfer of activated STING triggered by RAB22A-mediated non-canonical autophagy promotes antitumor immunity
Source: Cell Res. 2022 Oct 24;32(12):1086–104. doi: 10.1038/s41422-022-00731-w (PMC9715632; doi:10.1038/s41422-022-00731-w)
Supplement: Supplementary file 4 — Supplementary Figure S4 [file 41422_2022_731_MOESM4_ESM.pdf]

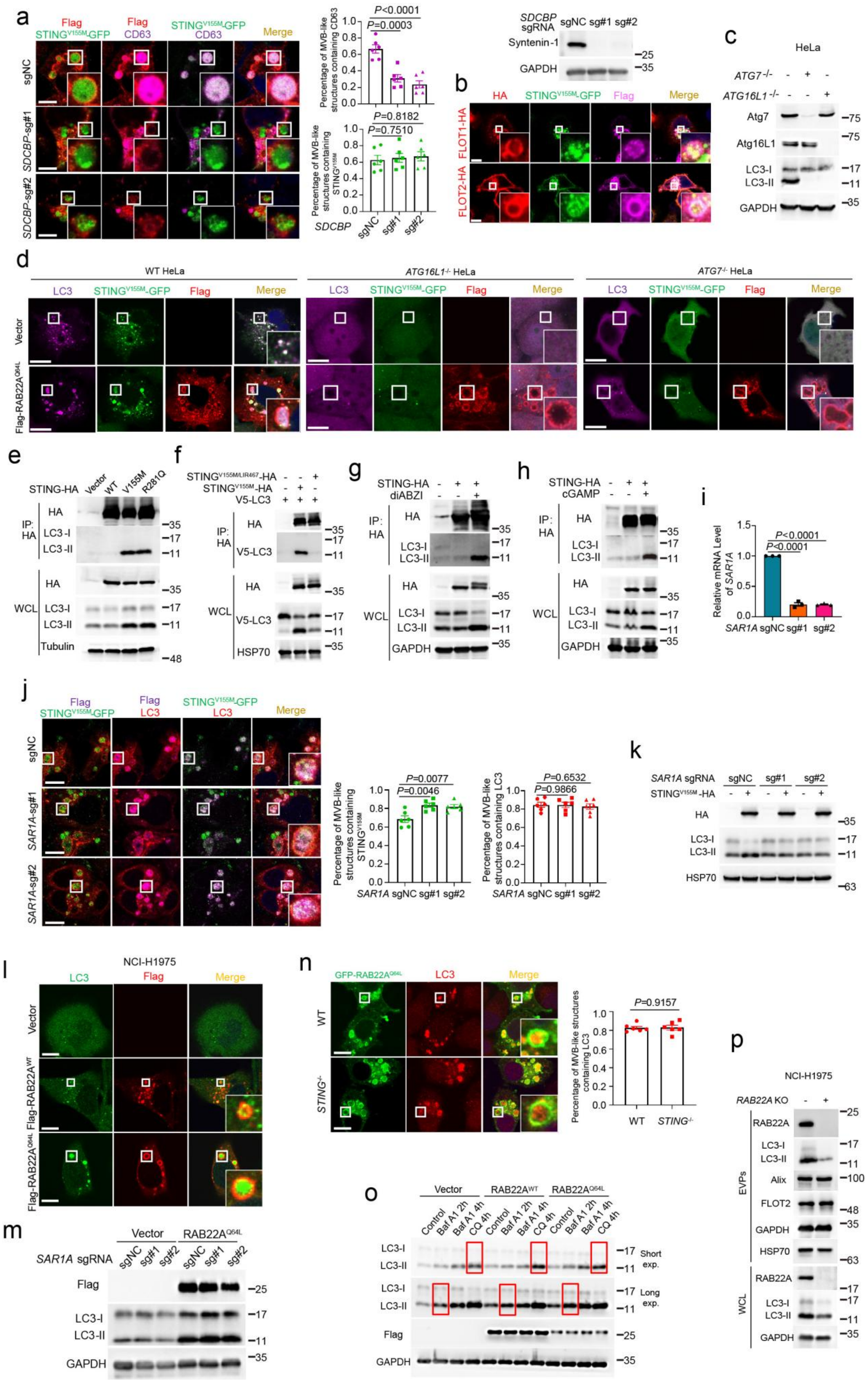
a
Flag STING^V155M-GFP
Flag CD63
STING^V155M-GFP CD63
Merge
sgNC
SDCBP-sg#1
SDCBP-sg#2
Percentage of MVB-like structures containing CD63
P<0.0001
P=0.0003
P=0.8182
P=0.7510
Percentage of MVB-like structures containing STING^V155M
SDCBP sgNC sg#1 sg#2
SDCBP sgRNA sgNC sg#1 sg#2
Syntenin-1
GAPDH
b
HA STING^V155M-GFP Flag Merge
FLOT1-HA
FLOT2-HA
c
HeLa
ATG7-/-
ATG16L1-/-
Atg7
Atg16L1
LC3-I
LC3-II
GAPDH
d
WT HeLa
ATG16L1-/- HeLa
ATG7-/- HeLa
LC3 STING^V155M-GFP Flag Merge
Vector
Flag-RAB22A^Q64L
e
STING-HA Vector WT V155M R281Q
IP: HA
WCL
HA
LC3-I
LC3-II
Tubulin
f
STING^V155M/LIR467-HA
STING^V155M-HA
V5-LC3
HSP70
g
STING-HA
diABZI
h
STING-HA
cGAMP
i
Relative mRNA Level of SAR1A
P<0.0001
P<0.0001
SAR1A sgNC sg#1 sg#2
j
Flag STING^V155M-GFP
Flag LC3
STING^V155M-GFP LC3
Merge
sgNC
SAR1A-sg#1
SAR1A-sg#2
P=0.0077
P=0.0046
Percentage of MVB-like structures containing STING^V155M
P=0.6532
P=0.9866
Percentage of MVB-like structures containing LC3
SAR1A sgNC sg#1 sg#2
k
SAR1A sgRNA sgNC sg#1 sg#2
STING^V155M-HA
HA
LC3-I
LC3-II
HSP70
l
NCI-H1975
LC3 Flag Merge
Vector
Flag-RAB22A^WT
Flag-RAB22A^Q64L
m
Vector RAB22A^Q64L
SAR1A sgRNA sgNC sg#1 sg#2 sgNC sg#1 sg#2
Flag
LC3-I
LC3-II
GAPDH
n
GFP-RAB22A^Q64L LC3 Merge
WT
STING-/-
P=0.9157
Percentage of MVB-like structures containing LC3
WT STING-/-
o
Vector RAB22A^WT RAB22A^Q64L
Control Baf A1 2h Baf A1 4h CQ 4h
LC3-I
LC3-II
Short exp.
Long exp.
Flag
GAPDH
p
NCI-H1975
RAB22A KO
EVPs
RAB22A
LC3-I
LC3-II
Alix
FLOT2
GAPDH
HSP70
WCL

Supplementary information, Fig. S4 The package of activated STING into MVB-like structures whose formation is driven by RAB22A is dependent on Atg5 but not on Syntenin-1, FLOTs, or SARA1.

a Knockout efficiency of *SDCBP* by Western blot (upper right) and localization of CD63 (magenta) and transiently expressing STING^{V155M}-GFP (green) in stably expressing Flag-RAB22A^{Q64L} (red) HeLa cells after *SDCBP* KO. Percentage of MVB-like structures containing CD63 and STING^{V155M} were quantified on the right. *P* values were calculated by student's *t*-test. *n* = 6 fields. Scale bar, 10 μm.

b Immunofluorescence analysis of FLOT1-HA (FLOT2-HA) (red) and Flag-RAB22A^{Q64L} (magenta) with STING^{V155M}-GFP (green) in stable Flag-RAB22A^{Q64L} HeLa cells transiently expressing STING^{V155M}-GFP and FLOT1-HA (FLOT2-HA). Scale bar, 10 μm.

c Knockout efficiency of *ATG7* and *ATG16L1* by Western blot.

d Immunofluorescence of LC3 (magenta), Flag-RAB22A^{Q64L} (red), DAPI (blue) and transiently transfected STING^{V155M}-GFP (green) in wide-type, *ATG7*$^{-/-}$ or *ATG16L1*$^{-/-}$ HeLa cells. Scale bar, 10 μm.

e Western blot analyses of whole-cell lysates (WCL) and immunoprecipitates from the indicated stable HeLa cells.

f Western blot analyses of WCL and immunoprecipitates from HEK-293T cells cotransfected with the indicated plasmids.

g, **h** Western blot analyses of WCL and immunoprecipitates from stable STING-HA-expressing HeLa cells treated with or without diABZI/cGAMP for 1 h. diABZI, 10 μM, cGAMP, 1 μM.

i QPCR-verified sgRNA knockout efficiency of *SARA1*. *P* values were calculated by student's *t*-test.

j Immunofluorescence analysis of LC3 (red) and Flag-RAB22A^{Q64L} (magenta) with STING^{V155M}-GFP (green) in the indicated stable HeLa cells transiently expressing STING^{V155M}-GFP. Percentage of MVB-like structures containing STING^{V155M} and LC3 were quantified on the upper right. *P* values were

calculated by student's *t*-test. *n* = 6 fields. Scale bar, 10 μm.

k Western blot analyses of whole-cell lysates (WCL) derived from the *SARA1*-KO HeLa cells transiently expressing $STING^{V155M}$-HA.

l Immunofluorescence analysis of LC3 (green) and Flag-$RAB22A^{Q64L}$ (red) in the indicated stable NCI-H1975 cells. Scale bar, 10 μm.

m Western blot analyses of WCL derived from the indicated stable HeLa cells.

n Immunofluorescence analysis of GFP-$RAB22A^{Q64L}$ (green) and LC3 (red) in the indicated HeLa cells. Percentage of MVB-like structures containing LC3 was quantified on the right. *P* values were calculated by student's *t*-test. *n* = 6 fields. Scale bar, 10 μm.

o Western blot analyses of WCL derived from 3× Flag-$RAB22A^{WT}$ or 3× Flag-$RAB22A^{Q64L}$ stable HeLa cells treated with 200 nM BafA1 or 10 μM CQ at the indicated time points.

p Western blot analyses of WCL and EVPs derived from *RAB22A*-KO NCI-H1975 cell.
