# Supplementary material for: Intercellular transfer of activated STING triggered by RAB22A-mediated non-canonical autophagy promotes antitumor immunity
Source: Cell Res. 2022 Oct 24;32(12):1086–104. doi: 10.1038/s41422-022-00731-w (PMC9715632; doi:10.1038/s41422-022-00731-w)
Supplement: Supplementary file 5 — Supplementary Figure S5 [file 41422_2022_731_MOESM5_ESM.pdf]

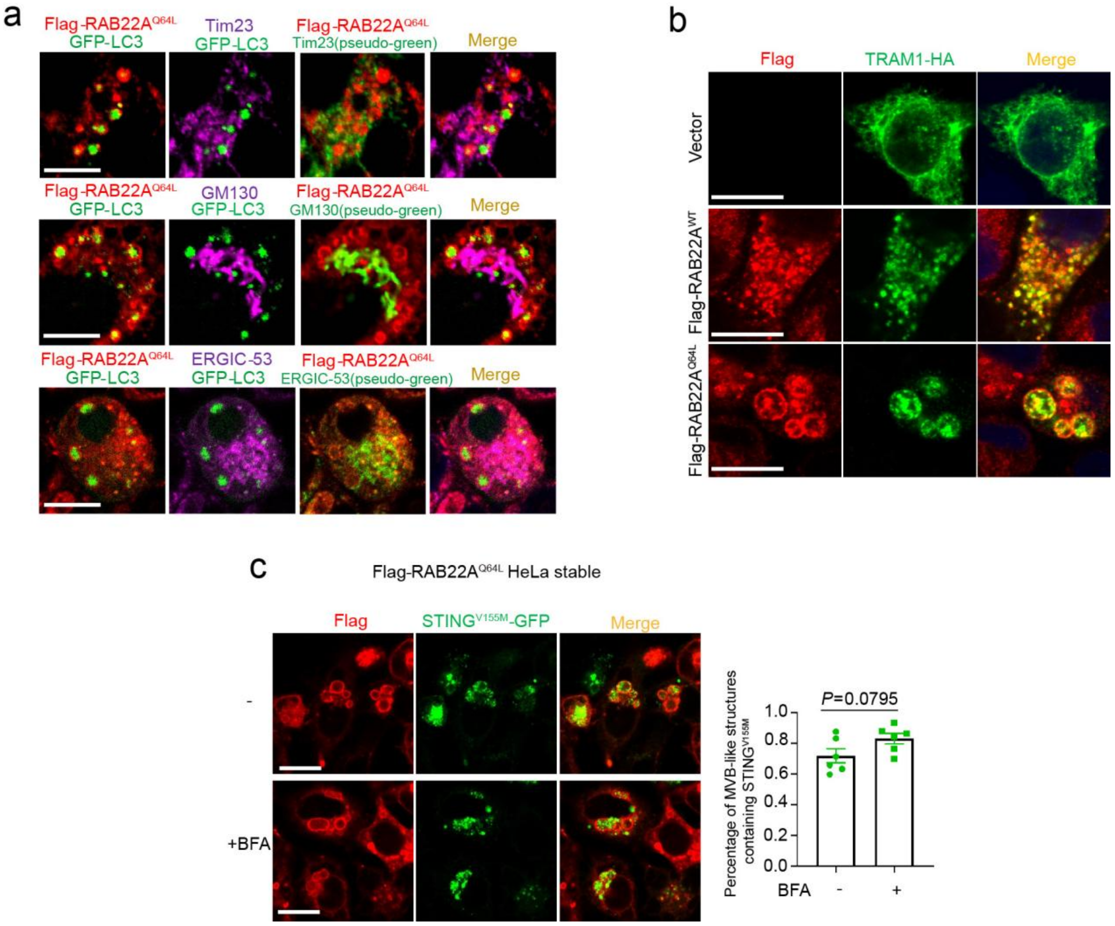

Supplementary information, Fig. S5 RAB22A-regulated non-canonical autophagosomes do not originate from mitochondria, the Golgi apparatus or the ER-Golgi intermediate compartment.

a Immunofluorescence analysis of the mitochondrial marker Tim23, Golgi apparatus marker GM130, and ER-Golgi intermediate compartment marker ERGIC-53 (magenta or pseudo-green as indicated) and Flag (red) with GFP-LC3 (green) in stable Flag-RAB22A^{Q64L} HeLa cells transiently expressing GFP-LC3. Scale bar, 10 μm.

b Immunofluorescence analysis of the ER marker TRAM1(green) and Flag (red) in stable Flag-RAB22A^{Q64L} HeLa cells transiently expressing TRAM1-HA. Scale bar, 10 μm.

c Immunofluorescence analysis of STING^{V155M}-GFP (green) and Flag-RAB22A^{Q64L} (red) in stable Flag-RAB22A^{Q64L} HeLa cells treated with or without 5 μg/mL BFA for 6 h. Percentage of MVB-like structures containing STING^{V155M}

was quantified on the right. *P* values were calculated by student's *t*-test. *n* = 6 fields. Scale bar, 10 μm.
