# Supplementary material for: Intercellular transfer of activated STING triggered by RAB22A-mediated non-canonical autophagy promotes antitumor immunity
Source: Cell Res. 2022 Oct 24;32(12):1086–104. doi: 10.1038/s41422-022-00731-w (PMC9715632; doi:10.1038/s41422-022-00731-w)
Supplement: Supplementary file 6 — Supplementary Figure S6 [file 41422_2022_731_MOESM6_ESM.pdf]

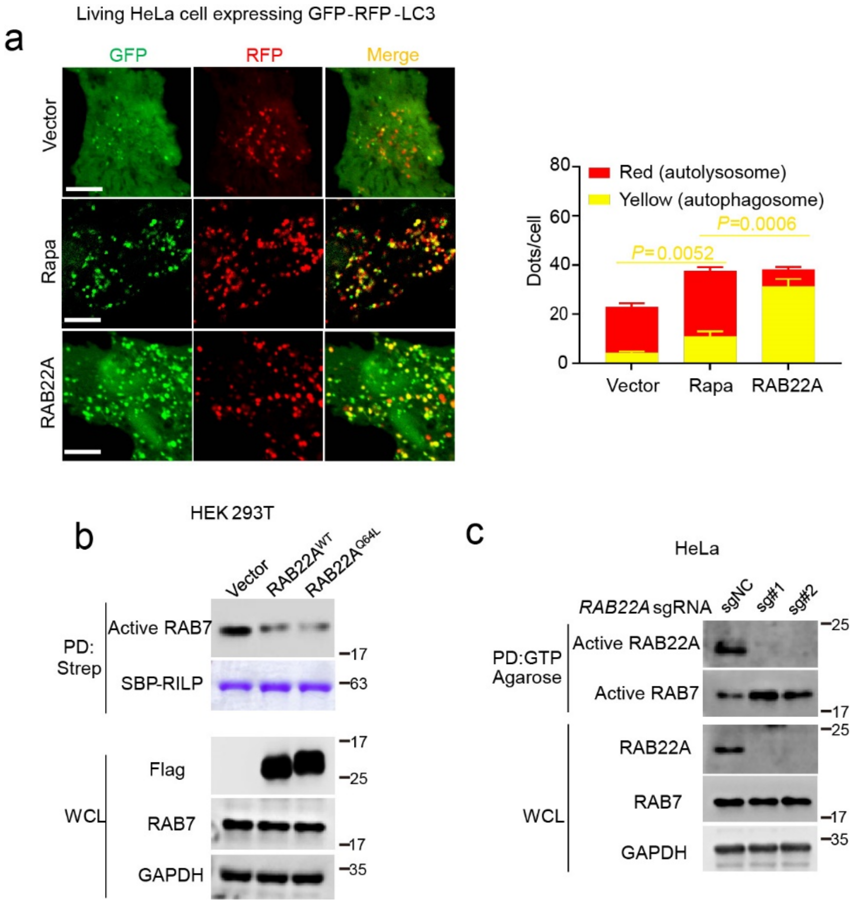

Supplementary information, Fig. S6 RAB22A inactivates RAB7, thereby enabling the secretion of inner vesicles of non-canonical autophagosomes as R-EVs from Rafeesomes.

a Vector-stable HeLa cells treated with or without Rapa (1 μM) for 6 h and RAB22A-stable HeLa cells transiently expressing GFP-RFP-LC3. The co-localization of GFP and RFP puncta in living cells was quantified. Puncta, either yellow with both green and red fluorescence (autolysosome) or red with only red fluorescence (autophagosome), were quantified (right panel). *P* values were calculated by student's *t*-test. Scale bar, 10 μm.

b Western blot analyses of whole-cell lysates (WCL) and streptavidin pull-down (PD) assay proteins obtained from HEK 293T cells co-expressing the indicated plasmids with SBP RILP.

c Western blot analyses of WCL and GTP agarose PDs from stable *RAB22A*-knockout HeLa cells.
