# Supplementary material for: Intercellular transfer of activated STING triggered by RAB22A-mediated non-canonical autophagy promotes antitumor immunity
Source: Cell Res. 2022 Oct 24;32(12):1086–104. doi: 10.1038/s41422-022-00731-w (PMC9715632; doi:10.1038/s41422-022-00731-w)
Supplement: Supplementary file 7 — Supplementary Figure S7 [file 41422_2022_731_MOESM7_ESM.pdf]

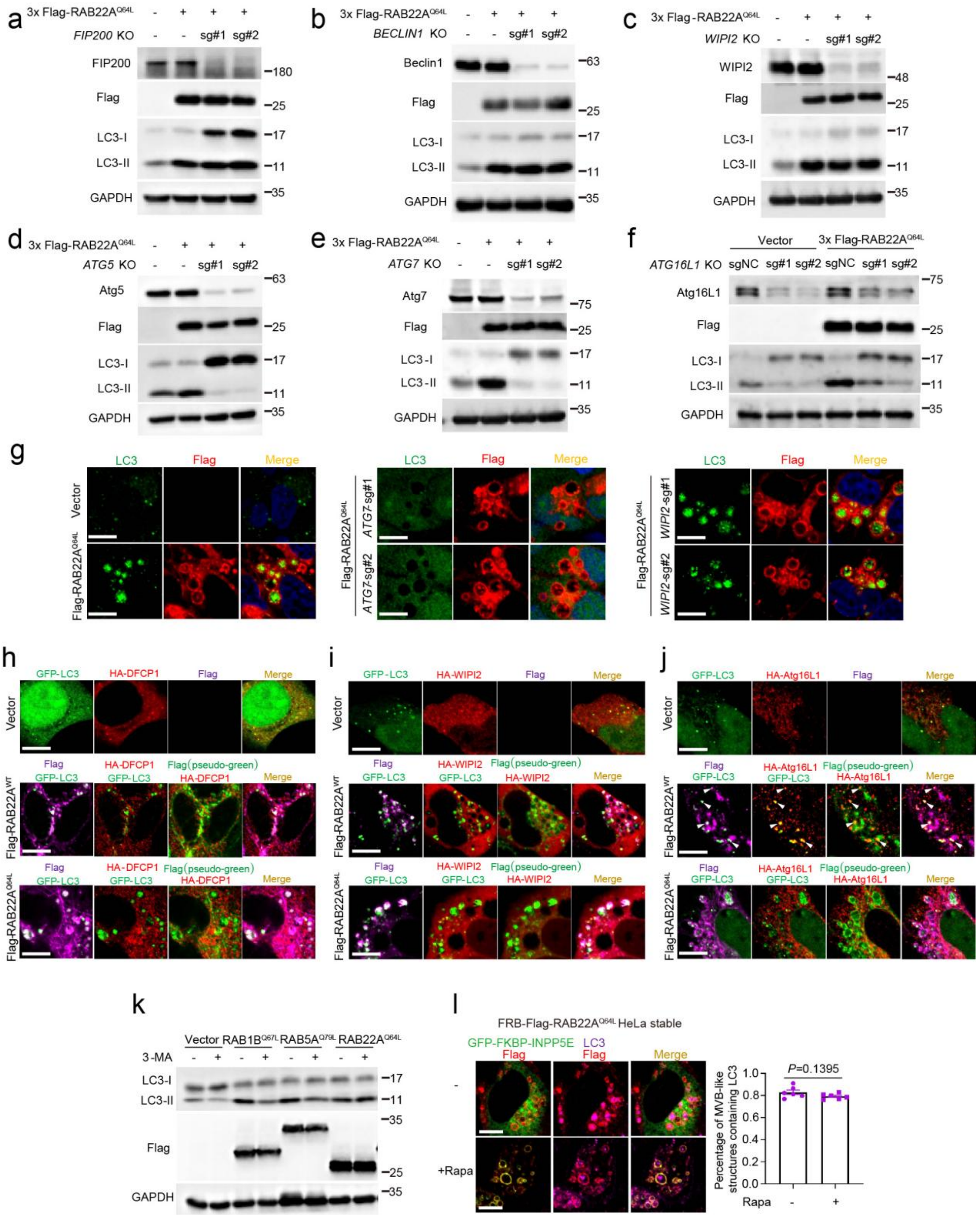
a
3x Flag-RAB22A Q64L - + + +
FIP200 KO - - sg#1 sg#2
FIP200
Flag
LC3-I
LC3-II
GAPDH
180
25
17
11
35
b
3x Flag-RAB22A Q64L - + + +
BECLIN1 KO - - sg#1 sg#2
Beclin1
Flag
LC3-I
LC3-II
GAPDH
63
25
17
11
35
c
3x Flag-RAB22A Q64L - + + +
WIPI2 KO - - sg#1 sg#2
WIPI2
Flag
LC3-I
LC3-II
GAPDH
48
25
17
11
35
d
3x Flag-RAB22A Q64L - + + +
ATG5 KO - - sg#1 sg#2
Atg5
Flag
LC3-I
LC3-II
GAPDH
63
25
17
11
35
e
3x Flag-RAB22A Q64L - + + +
ATG7 KO - - sg#1 sg#2
Atg7
Flag
LC3-I
LC3-II
GAPDH
75
25
17
11
35
f
Vector
3x Flag-RAB22A Q64L
ATG16L1 KO sgNC sg#1 sg#2 sgNC sg#1 sg#2
Atg16L1
Flag
LC3-I
LC3-II
GAPDH
75
25
17
11
35
g
LC3
Flag
Merge
Vector
Flag-RAB22A Q64L
ATG7-sg#1
ATG7-sg#2
WIPI2-sg#1
WIPI2-sg#2
h
GFP-LC3
HA-DFCP1
Flag
Merge
Vector
Flag-RAB22A WT
Flag-RAB22A Q64L
Flag (pseudo-green)
i
HA-WIPI2
j
HA-Atg16L1
k
Vector RAB1B Q67L RAB5A Q79L RAB22A Q64L
3-MA - + - + - + - +
LC3-I
LC3-II
Flag
GAPDH
17
11
35
25
35
l
FRB-Flag-RAB22A Q64L HeLa stable
GFP-FKBP-INPP5E
LC3
Flag
Merge
-
+Rapa
P=0.1395
Percentage of MVB-like structures containing LC3
1.0
0.8
0.6
0.4
0.2
0.0
Rapa
-
+

Supplementary information, Fig. S7 RAB22A-regulated non-canonical autophagy is dependent on Atg5, Atg7 and Atg16L1 but not FIP200, Beclin1, WIPI2.

a-f Western blot analyses of whole-cell lysates from stable 3× Flag-RAB22A^{Q64L} HeLa cells with *FIP200* (**a**), *BECLIN1* (**b**), *WIPI2* (**c**), *ATG5* (**d**), *ATG7* (**e**), and *ATG16L1* (**f**) knocked out using sgRNAs.

g Immunofluorescence analysis of Flag (red), LC3 (green) and DAPI (blue) in Flag-RAB22A^{Q64L} HeLa cells stably expressing *ATG7* or *WIPI2* sgRNAs. Scale bar, 10 μm.

h Immunofluorescence analysis of Flag (magenta or pseudo-green as indicated) and GFP-LC3 (green) with HA-DFCP1 (red) in the indicated stable HeLa cells transiently co-expressing GFP-LC3 and HA-DFCP1. Scale bar, 10 μm.

i Immunofluorescence analysis of Flag (magenta or pseudo-green as indicated) and GFP-LC3 (green) with HA-WIPI2 (red) in the indicated stable HeLa cells transiently co-expressing GFP-LC3 and HA-WIPI2. Scale bar, 10 μm.

j Immunofluorescence analysis of Flag (magenta or pseudo-green as indicated) and GFP-LC3 (green) with HA-Atg16L1 (red) in the indicated stable HeLa cells transiently co-expressing GFP-LC3 and HA-Atg16L1. Scale bar, 10 μm.

k Western blot analyses of whole-cell lysates from the indicated stable HeLa cells treated with or without 10 mM 3-MA for 6 h.

l Immunofluorescence analysis of Flag (red) and LC3 (magenta) with GFP-FKBP-INPP5E (green) in FRB-Flag-RAB22A^{Q64L} stable HeLa cells treated with or without 1 μM Rapa for 6 h. Percentage of MVB-like structures containing LC3 was quantified on the right. *P* values were calculated by student's *t*-test. *n* = 6 fields. Scale bar, 10 μm.
