# Supplementary material for: Intercellular transfer of activated STING triggered by RAB22A-mediated non-canonical autophagy promotes antitumor immunity
Source: Cell Res. 2022 Oct 24;32(12):1086–104. doi: 10.1038/s41422-022-00731-w (PMC9715632; doi:10.1038/s41422-022-00731-w)
Supplement: Supplementary file 8 — Supplementary Figure S8 [file 41422_2022_731_MOESM8_ESM.pdf]

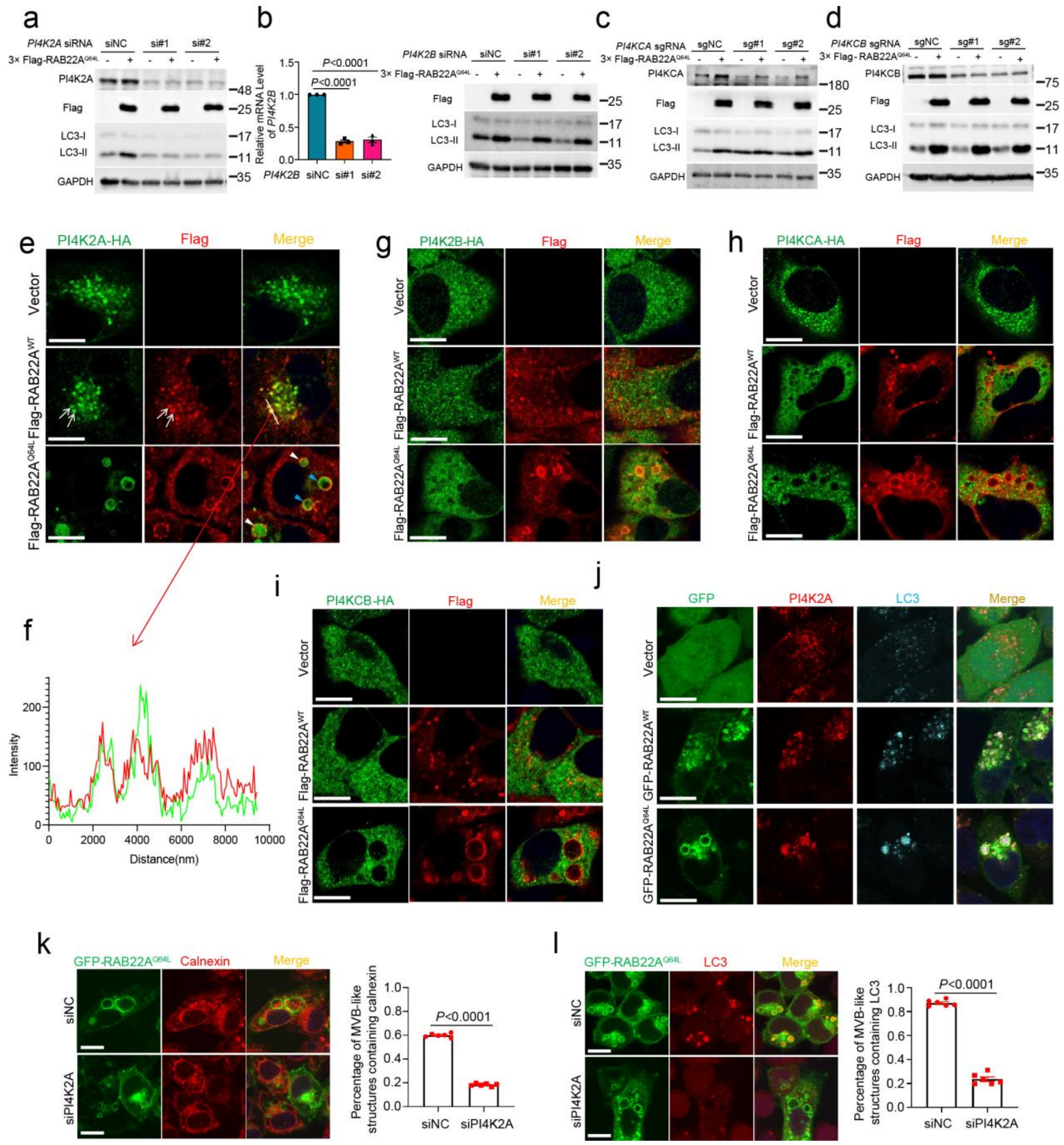
a
PI4K2A siRNA siNC si#1 si#2
3× Flag-RAB22A^{Q64L}
PI4K2A
Flag
LC3-I
LC3-II
GAPDH
b
P<0.0001
P<0.0001
Relative mRNA Level of PI4K2B
PI4K2B siNC si#1 si#2
PI4K2B siRNA siNC si#1 si#2
c
PI4KCA sgRNA sgNC sg#1 sg#2
PI4KCA
d
PI4KCB sgRNA sgNC sg#1 sg#2
PI4KCB
e
PI4K2A-HA Flag Merge
Vector
Flag-RAB22A^{WT}
Flag-RAB22A^{Q64L}
f
Intensity
Distance(nm)
g
PI4K2B-HA Flag Merge
h
PI4KCA-HA Flag Merge
i
PI4KCB-HA Flag Merge
j
GFP PI4K2A LC3 Merge
GFP-RAB22A^{WT}
GFP-RAB22A^{Q64L}
k
GFP-RAB22A^{Q64L} Calnexin Merge
siNC
siPI4K2A
Percentage of MVB-like structures containing calnexin
P<0.0001
l
GFP-RAB22A^{Q64L} LC3 Merge
Percentage of MVB-like structures containing LC3
P<0.0001

Supplementary information, Fig. S8 RAB22A-regulated non-canonical autophagy is dependent on PI4K2A but not PI4K2B, PI4KCA or PI4KCB.

a-d Verification of the efficiency of siRNAs targeting PI4K2B by qPCR (**b**). The data represent the means ± SEM. Western blot analyses of whole-cell lysates from stable 3× Flag-RAB22A^{Q64L} HeLa cells transfected with two different siRNAs targeting *PI4K2A* (**a**) or *PI4K2B* (**b**) or knockout of *PI4KCA* (**c**) or *PI4KCB* (**d**) using sgRNAs.

e-i Immunofluorescence analysis of Flag (red) and HA (green) in the indicated stable HeLa cells transiently expressing PI4K2A-HA (**e-f**), PI4K2B-HA (**g**), PI4KCA-HA (**h**), or PI4KCB-HA (**i**). White arrows denote co-localization; white arrowheads denote co-localization inside Rafeesomes; blue arrowheads denote co-localization on the surface of Rafeesomes. Scale bars, 10 μm.

j Immunofluorescence analysis of GFP (green), PI4K2A (red) and LC3 (cyan) in the indicated stable HeLa cells. Scale bar, 10 μm.

k Immunofluorescence analysis of GFP-RAB22A^{Q64L} (green) and calnexin (red) in the indicated HeLa cells transfected with siRNAs targeting PI4K2A. Percentage of MVB-like structures containing calnexin was quantified on the right. *P* values were calculated by student's *t*-test. *n* = 6 fields. Scale bar, 10 μm.

l Immunofluorescence analysis of GFP-RAB22A^{Q64L} (green) and LC3 (red) in the indicated HeLa cells transfected with siRNAs targeting PI4K2A. Percentage of MVB-like structures containing LC3 was quantified on the right. *P* values were calculated by student's *t*-test. *n* = 6 fields. Scale bar, 10 μm.
