# Supplementary material for: Intercellular transfer of activated STING triggered by RAB22A-mediated non-canonical autophagy promotes antitumor immunity
Source: Cell Res. 2022 Oct 24;32(12):1086–104. doi: 10.1038/s41422-022-00731-w (PMC9715632; doi:10.1038/s41422-022-00731-w)
Supplement: Supplementary file 9 — Supplementary Figure S9 [file 41422_2022_731_MOESM9_ESM.pdf]

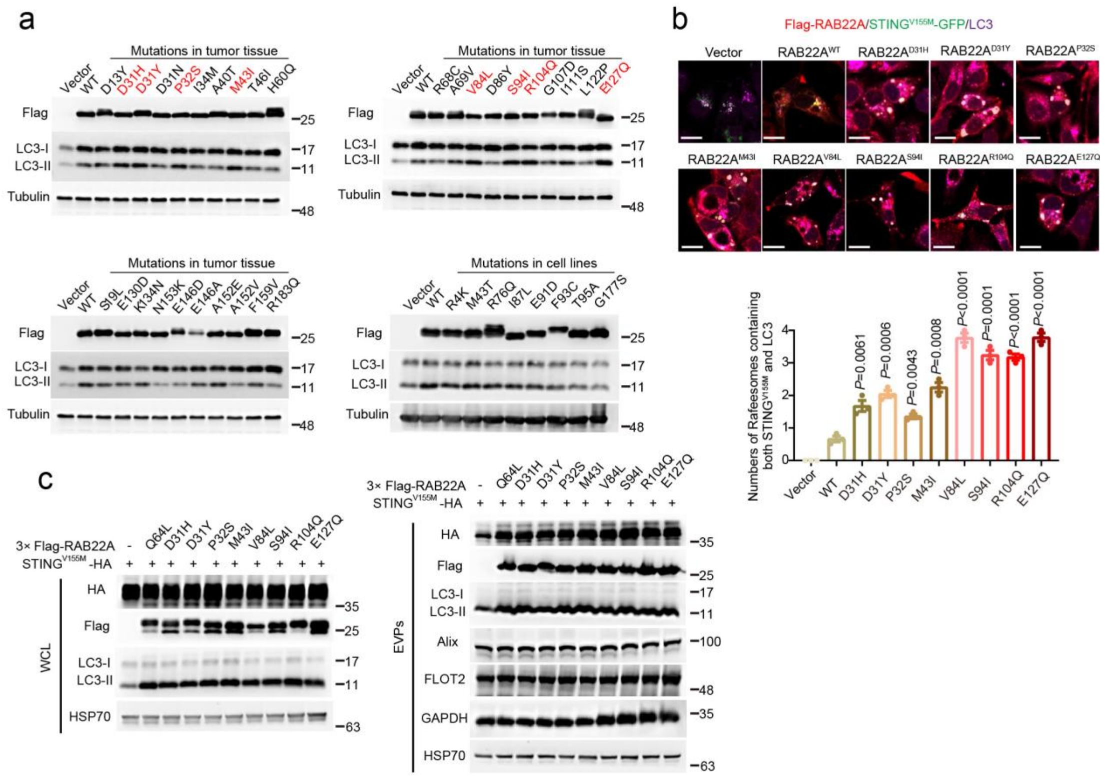

Supplementary information, Fig. S9 Some RAB22A mutants detected in human cancers could increase LC3-II and promote active STING to be secreted into EVs.

a Western blot analyses of the whole-cell lysates (WCL) of the indicated RAB22A mutation stable HeLa cells.

b Immunofluorescence analysis of STING^{V155M}-GFP (green) and Flag-RAB22A^{Q64L} (red) with GFP-LC3 (magenta) in the indicated RAB22A mutant stable HeLa cells transiently expressing STING^{V155M}-GFP. Numbers of Rafeesomes containing both STING and LC3 were quantified as below. *P* values were calculated by student's *t*-test. *n* = 6 fields. Scale bar, 10 μm.

c Western blot analyses of the WCL and EVPs of the indicated RAB22A mutation stable HeLa cells with overexpressing STING^{V155M}-HA.
