# Supplementary material for: Intercellular transfer of activated STING triggered by RAB22A-mediated non-canonical autophagy promotes antitumor immunity
Source: Cell Res. 2022 Oct 24;32(12):1086–104. doi: 10.1038/s41422-022-00731-w (PMC9715632; doi:10.1038/s41422-022-00731-w)
Supplement: Supplementary file 10 — Supplementary Table S1 [file 41422_2022_731_MOESM10_ESM.pdf]

Table S1: sgRNA sequences for CRISPR knock out.

Genes	sgRNA sequence 1	sgRNA sequence 2
cGAS	GCCGCCGTGGAGATATCATCG	
STING	CATATTACATCGGATATCTG	
RAB22A	TAGGTAAATCGAGTATTGTG	AGTCATGGAGAGAGATGCAA
SDCBP	AAGTGGTGCACCAGAAACCA	AATGGACCACACCATTCCTG
SAR1A	GCTCAACTTTGGATTCCACG	GATGTAGTGTTGGAACATGT
ATG5	GATGTAGTGTTGGAACATGT	GCCAACTCCACCTTGCCCGG
ATG7	ACAAGCCCAAGAGAGGTTGG	CAAACCGTGAAAGAAATCCC
ATG16L1	CACACACTCACGGGACACAG	ACAGAGCAATGTGTAATGAG
FIP200	CATGCCGGGAAACGGCCTGC	AGTTCACAGTCAAACTTTCG
BECLIN1	CCAAGTCCGGTCTACCGCGG	CACCCAAGTCCGGTCTACCG
WIPI2	GAGGCTGATAGTATGCCTGG	CATGTACAGGTACCCGTCGG
PI4KCA	GAGAAAGAAGGCTTGTCTGT	TGTCCAACATGAGAGTGACC
PI4KCB	GCACGGCAGTTACACCACTG	GGATGAAGCCAAAGTCGATG
