# Supplementary material for: Intercellular transfer of activated STING triggered by RAB22A-mediated non-canonical autophagy promotes antitumor immunity
Source: Cell Res. 2022 Oct 24;32(12):1086–104. doi: 10.1038/s41422-022-00731-w (PMC9715632; doi:10.1038/s41422-022-00731-w)
Supplement: Supplementary file 11 — Supplementary Table S2 [file 41422_2022_731_MOESM11_ESM.pdf]

Table S2: shRNA sequences for knock down.

Genes	shRNA sequence 1	shRNA sequence 2
Mouse PI4K2A	CCGTTCTCTCAGGAGATCAAA	CCCAAGAATGAAGAGCCATAT
