# Supplementary material for: Intercellular transfer of activated STING triggered by RAB22A-mediated non-canonical autophagy promotes antitumor immunity
Source: Cell Res. 2022 Oct 24;32(12):1086–104. doi: 10.1038/s41422-022-00731-w (PMC9715632; doi:10.1038/s41422-022-00731-w)
Supplement: Supplementary file 12 — Supplementary Table S3 [file 41422_2022_731_MOESM12_ESM.pdf]

Table S3: Primers for qPCR.

Genes	Forward Primers	Reverse Primers
GAPDH	GGAGCGAGATCCCTCCAAAAT	GGCTGTTGTCATACTTCTCATGG
IFNβ	ATGACCAACAAGTGTCTCCTCC	GGAATCCAAGCAAGTTGTAGCTC
SAR1A	CAACACTACATCCGACATCAGAA	TCCACGAGGCGAGAATGATCT
PI4K2B	ACCCAAATCAGAAGAGCCTTATG	CAAGGGCAGCAGACCTTATG
