# Supplementary material for: Intercellular transfer of activated STING triggered by RAB22A-mediated non-canonical autophagy promotes antitumor immunity
Source: Cell Res. 2022 Oct 24;32(12):1086–104. doi: 10.1038/s41422-022-00731-w (PMC9715632; doi:10.1038/s41422-022-00731-w)
Supplement: Supplementary file 13 — Supplementary video legend [file 41422_2022_731_MOESM13_ESM.pdf]

Supplementary information, video S1: Time-lapse images of GFP-RAB22A^{Q64L} (green) and calnexin-mCherry (red) in tet-on GFP-RAB22A^{Q64L} stable living HeLa cells transiently expressing calnexin-mCherry treated with 50 ng/mL doxycycline (dox).

Supplementary information, video S2: Time-lapse images of GFP-RAB22A^{Q64L} (green) and STING^{V155M}-Halo (red) in tet-on GFP-RAB22A^{Q64L} stable living HeLa cells transiently expressing STING^{V155M}-Halo and treated with 50 ng/mL dox.
